# Development of a dose-limiting data collection strategy for serial synchrotron rotation crystallography

**DOI:** 10.1107/S1600577516016362

**Published:** 2017-01-01

**Authors:** Kazuya Hasegawa, Keitaro Yamashita, Tomohiro Murai, Nipawan Nuemket, Kunio Hirata, Go Ueno, Hideo Ago, Toru Nakatsu, Takashi Kumasaka, Masaki Yamamoto

**Affiliations:** a Japan Synchrotron Radiation Research Institute, 1-1-1 Kouto, Sayo 679-5198, Japan; b RIKEN SPring-8 Center, 1-1-1 Kouto, Sayo 679-5148, Japan; c Graduate School of Pharmaceutical Sciences, Kyoto University, Sakyo, Kyoto 606-8501, Japan; d Japan Science and Technology Agency, Precursory Research for Embryonic Science and Technology (PRESTO), 4-1-8 Honcho, Kawaguchi, Saitama 332-0012, Japan

**Keywords:** serial synchrotron crystallography, mercury single-wavelength anomalous diffraction, radiation damage

## Abstract

Serial crystallography, in which single-shot diffraction images are collected, has great potential for protein microcrystallography. Although serial femtosecond crystallography (SFX) has been successfully demonstrated, limited beam time prevents its routine use. Inspired by SFX, serial synchrotron crystallography (SSX) has been investigated at synchrotron macromolecular crystallography beamlines. Unlike SFX, the longer exposure time of milliseconds to seconds commonly used in SSX causes radiation damage. However, in SSX, crystals can be rotated during the exposure, which can achieve efficient coverage of the reciprocal space. In this study, mercury single-wavelength anomalous diffraction (Hg-SAD) phasing of the luciferin regenerating enzyme (LRE) was performed using serial synchrotron rotation crystallography. The advantages of rotation and influence of dose on the data collected were evaluated. The results showed that sample rotation was effective for accurate data collection, and the optimum helical rotation step depended on multiple factors such as multiplicity and partiality of reflections, exposure time per rotation angle and the contribution from background scattering. For the LRE microcrystals, 0.25° was the best rotation step for the achievable resolution limit, whereas a rotation step larger than or equal to 1° was favorable for Hg-SAD phasing. Although an accumulated dose beyond 1.1 MGy caused specific damage at the Hg site, increases in resolution and anomalous signal were observed up to 3.4 MGy because of a higher signal-to-noise ratio.

## Abstract 2

The best practice for dose-limiting serial synchrotron rotation crystallography was examined through anomalous signal and single-wavelength anomalous diffraction phasing of mercury-bound luciferin regenerating enzyme. Sample rotation enabled accurate data collection with fewer diffraction images than without rotation, and an increase in resolution and anomalous signal was observed up to 3.4 MGy even though specific damage occurred after an accumulated dose of 1.1 MGy.

## 1. Introduction   

Macromolecular crystallography (MX) has contributed to the structure determination of more than 109000 proteins as of September 2016 (http://www.wwpdb.org), but there are still many challenging and scientifically important targets yet to have their structure solved. One of the difficulties in structure determination of these targets is obtaining large, well diffracting crystals. To cope with smaller samples, synchrotron microfocus beamlines have been developed (Riekel *et al.*, 2005 ▸; Fischetti *et al.*, 2009 ▸; Flot *et al.*, 2010 ▸; Smith *et al.*, 2012 ▸; Hirata *et al.*, 2013 ▸). A microbeam with a photon flux density of 10^10^ to 10^11^ photons s^−1^ µm^−2^ not only increases the diffraction intensity but also decreases the background scattering from the surrounding mother liquor or buffer.

However, great care is needed when using a microbeam because radiation damage causes diffraction data quality to deteriorate (Holton, 2009 ▸; Garman, 2010 ▸). Henderson (1990 ▸) estimated that the diffraction intensity halves after accumulation of 20 MGy during MX data collection at cryogenic temperatures. Owen *et al.* (2006 ▸) experimentally determined a recommended dose limit of 30 MGy, at which the diffraction intensity of apoferritin and holoferritin crystals decreased to 70% of the initial value. They showed that biological information obtained from the structure beyond this limit is compromised. Liebschner *et al.* (2015 ▸) reported that the diffraction intensity of thaumatin crystals decreased to 70% of the initial value upon accumulation of about 10 MGy, and suggested that the dose limit depended on the type of protein crystal. These dose limits force a compromise between attainable resolution and completeness/multiplicity. Radiation damage also affects the local structure of proteins, such as breakage of disulfide bonds and decarboxylation of glutamates and aspartates (Weik *et al.*, 2000 ▸; Burmeister, 2000 ▸; Ravelli & McSweeney, 2000 ▸). The heavy atoms bound to proteins are especially susceptible to damage because they possess larger photoelectric cross sections than those of lighter atoms. Such local damage is known to affect experimental phasing using heavy atoms (Schiltz *et al.*, 2004 ▸; Ramagopal *et al.*, 2005 ▸; Ravelli *et al.*, 2005 ▸; González, 2007 ▸). Damage to local sites progresses faster than global damage and its rate depends on various factors such as the species damaged and the environment of the damaged site (Holton, 2009 ▸; Garman, 2010 ▸).

One approach to decrease the effect of radiation damage is to translate the crystal during helical data collection, which is applicable when the beam is smaller than the crystal (Flot *et al.*, 2010 ▸). By translating the crystal, fresh crystal volume is exposed to the beam to reduce the overall dose and thus partially mitigate the influence of radiation damage. When the crystal is too small to translate, it is difficult to obtain complete data from one crystal. In these cases, a multicrystal data collection strategy is useful, in which data are completed by merging small wedges collected from multiple crystals. This approach has been automated by combining raster scanning to identify suitable crystal positions and subsequent partial data collection (Zander *et al.*, 2015 ▸; Hirata *et al.*, 2017 ▸). However, this strategy still has drawbacks; the number of images that can be collected from one crystal is limited when the crystal is small. Moreover, a high dose is needed to identify suitable positions of weakly diffracting crystals, which can lead to severe damage even prior to full data collection.

Serial femtosecond crystallography (SFX) at X-ray free-electron lasers (XFELs) (Chapman *et al.*, 2011 ▸) is another approach based on the principle of ‘diffraction before destruction’ (Neutze *et al.*, 2000 ▸); the pulse duration of a few to a few tens of femtoseconds used in SFX enables collection of a diffraction pattern before the protein structure is destroyed by a Coulomb explosion (Boutet *et al.*, 2012 ▸). To date, the performance of SFX in microcrystallography has been exemplified by successful *de novo* phasing (Barends *et al.*, 2014 ▸; Yamashita *et al.*, 2015 ▸; Nakane *et al.*, 2015 ▸; Fukuda *et al.*, 2016 ▸; Nass *et al.*, 2016 ▸) as well as structure determination using molecular replacement (Chapman *et al.*, 2011 ▸; Boutet *et al.*, 2012 ▸; Redecke *et al.*, 2013 ▸; Liu *et al.*, 2013 ▸; Weierstall *et al.*, 2014 ▸; Kern *et al.*, 2014 ▸; Kupitz *et al.*, 2014 ▸; Tenboer *et al.*, 2014 ▸). However, the limited availability of XFEL beam time prevents SFX from becoming a general method for protein microcrystallography. Moreover, with the current technique, a huge number of diffraction patterns are required for accurate data collection. For example, single-wavelength anomalous diffraction (SAD) phasing using a gadolinium derivative of lysozyme required 60000 indexed patterns (Barends *et al.*, 2014 ▸), which was recently lowered to 7000 by an improvement in the data processing (Nass *et al.*, 2016 ▸).

Inspired by the success of SFX, serial synchrotron crystallography (SSX) has been examined at synchrotron MX beamlines (Gati *et al.*, 2014 ▸; Stellato *et al.*, 2014 ▸; Nogly *et al.*, 2015 ▸; Botha *et al.*, 2015 ▸; Coquelle *et al.*, 2015 ▸). Unlike in SFX, the exposure time of milliseconds to seconds in SSX can cause radiation damage; however, it has the advantage that the crystals can be rotated during exposure, which increases the accuracy and efficiency of data collection (Huang *et al.*, 2015 ▸, 2016 ▸). Gati *et al.* (2014 ▸) collected diffraction data from loop-harvested microcrystals of *Tb*CatB by two-dimensional raster scanning combined with goniometer rotation. They solved the structure by molecular replacement and demonstrated the capability of serial synchrotron rotation crystallography (SS-ROX) for protein microcrystallography with tolerable radiation damage. Inspired by their work, here we report the Hg-SAD phasing of luciferin regenerating enzyme (LRE) using SS-ROX. To demonstrate the prospects and limitations of serial microcrystallography at synchrotron beamlines, we evaluate the effects of rotation angle per image on resolution and data quality, and verify the influence of dose on the anomalous signal and phasing. Unlike Gati *et al.* (2014 ▸), rather than grouping and processing together consecutive images from the same crystal, we processed all the images as random snapshots. Our results show that sample rotation is effective for accurate data collection. Larger rotation steps enable Hg-SAD phasing with fewer images, but the optimum rotation step depends on the balance between the multiplicity and partiality of reflections, exposure time per rotation angle, and the contribution from background scattering. Although there is specific damage at the Hg site when the accumulated dose exceeds 1.1 MGy, increases in resolution and anomalous signal intensity are observed up to 3.4 MGy because of a higher signal-to-noise ratio (S/N).

## 2. Material and methods   

### 2.1. Sample preparation   

Microcrystals of mercury-bound LRE consisting of 308 amino acid residues were used. The crystals belonged to the space group 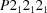 with unit-cell parameters *a* = 47.3, *b* = 76.7, *c* = 84.0 Å, and contained one monomer per asymmetric unit. There was one major and one minor mercury site in the vicinity of Cys52. Yamashita *et al.* (2015 ▸) have previously described the protocol for the preparation of LRE microcrystals and their mercury derivative. The LRE microcrystals were rod shaped with a thickness of 3–5 µm and length of 20–50 µm. The LRE microcrystals were mounted on LithoLoops (Protein Wave Co., Japan) with a diameter of 1.0 mm and flash cooled under a cryo stream operating at 107 K. The average thickness of the sample at the center of a loop was 0.12 mm (0.02 r.m.s.d.).

### 2.2. Data collection with SS-ROX   

Data collection was conducted at SPring-8 BL41XU using a wavelength of 0.9839 Å, which was the same wavelength as that used during a SFX experiment at SACLA (Yamashita *et al.*, 2015 ▸, 2017 ▸). A microbeam of 5 µm (vertical; V) × 4 µm (horizontal; H) (full width at half-maximum) with a photon flux of 2.2–2.4 × 10^12^ photons s^−1^ was used. The samples were kept at 107 K under a cryo nitrogen flow using CRYOCOOL-G2b-LT (Cryo Industries of America, Inc., USA). Diffraction images were recorded with a PILATUS3 6M detector (Dectris Ltd, Switzerland).

**Figure 1:**
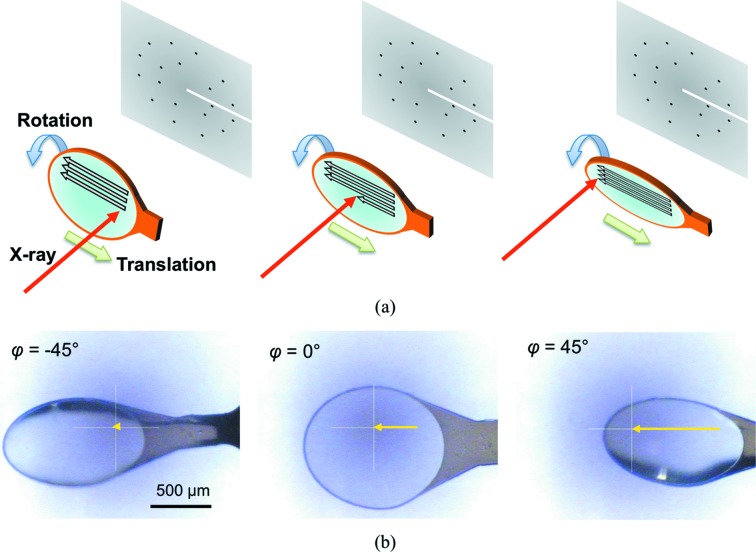
Overview of SS-ROX data collection. (*a*) Schematic diagrams of two-dimensional raster scans with goniometer rotation. The loop on which the crystals were loaded was raster-scanned with rotation of the goniometer spindle axis. (*b*) Photographs of a horizontal helical scan; yellow arrows represent relative movement of the incident X-ray position. φ is the goniometer spindle angle.

The loop on which the crystals were loaded was raster-scanned with rotation of the goniometer spindle axis (Fig. 1 ▸). The spindle was rotated as the crystal was being translated. An area of 750 µm (V) × 720 µm (H) on the loop was scanned by 75 horizontal helical scans with a vertical interval of 10 µm between neighboring scans. A total of 180 images were recorded in each horizontal helical scan with a horizontal translation of 4 µm between each image. This translation length was identical to the beam width. The rotation and exposure time per image were changed as described below.

#### 2.2.1. Data collection using different helical rotation steps   

To examine the influence of the helical rotation step on the data, we collected seven datasets using a helical rotation step per image (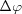) of 0.0°, 0.1°, 0.25°, 0.5°, 1.0°, 1.5° and 2.0°; these datasets are labeled Rs-0, Rs-0.1, Rs-0.25, Rs-0.5, Rs-1, Rs-1.5 and Rs-2, respectively. The total goniometer rotation range for each helical scan was ±0° for Rs-0, ±9° for Rs-0.1, ±22.5° for Rs-0.25 and ±45° for Rs-0.5, where 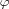 = 0° is the angle where the loop surface is perpendicular to the incident beam. For Rs-1, to prevent the scan range from being ±90°, the scan area was divided into two areas of 750 µm (V) × 360 µm (H) and the number of images per helical line was halved. This decreased the scan range to ±45°. In a similar way, the scan areas of both Rs-1.5 and Rs-2 were divided into three and four, respectively, and each area was scanned for a rotation of ±45°. All datasets were collected with a frame rate of 50 frames s^−1^, which corresponds to an exposure time of 0.02 s frame^−1^. The number of loops used for data collection was five for Rs-0 and Rs-0.1, four for Rs-0.25 and two for the other datasets. The total number of collected images for each dataset is shown in Table 1 ▸.

#### 2.2.2. Data collection using different doses   

The influence of dose on the data was examined by collecting seven datasets using different exposure times of 0.02, 0.04, 0.0556, 0.08, 0.111, 0.222 and 0.435 s frame^−1^, which corresponds to doses of 1.2, 2.4, 3.4, 4.8, 6.7, 13 and 26 MGy, respectively; these datasets are labeled Da-1.2, Da-2.4, Da-3.4, Da-4.8, Da-6.7, Da-13 and Da-26, respectively. To trace the damage caused by accumulating dose, we also performed 25 scans on the same sample with an exposure time of 0.02 s, which corresponds to 1.1 MGy; these datasets are labeled Ds-1.1^*i*th^ (*i* = 1, 2,…, 25). All datasets here were collected using 0.5° frame^−1^.

The dose was estimated using *RADDOSE-3D* (Zeldin *et al.*, 2013 ▸), assuming a uniform beam profile of 5 µm (V) × 4 µm (H) and crystal dimensions of 4 µm × 4 µm × 30 µm. The helical scan that completely went across a crystal was simulated in the calculation. Photon fluxes of 2.4 × 10^12^ and 2.3 × 10^12^ (photons s^−1^) were used for Da-1.3 to Da-26 and Ds-1.1^*i*th^ (*i* = 1, 2,…, 25), respectively. Among several dose-based metrics calculated by *RADDOSE-3D*, we used the average dose-exposed region (AD-ER) (Zeldin *et al.*, 2013 ▸) as a metric for the absorbed dose. The accumulated dose for each crystal after data collection is equal to the estimated value. However, this is an expected maximum dose per frame because there is a possibility that the X-rays started to hit the crystal in the middle of the exposure of a frame and the crystal was not totally bathed in the beam at the beginning of the exposure.

### 2.3. Data processing and Hg-SAD phasing   

#### 2.3.1. Hit finding   

We adapted the peak finding function of *Cheetah* (Barty *et al.*, 2014 ▸) for hit finding. In peak finding, integrated intensity was evaluated using background based on radial average. An image was considered a hit if three or more spots were found in the area with resolution lower than 5.0 Å. The hit images were subjected to data processing using *CrystFEL* (White *et al.*, 2016 ▸) and *XDS* (Kabsch, 2010*b* ▸). The modified version of *Cheetah* (*cheetah.local_singles*) is available on GitHub (https://github.com/keitaroyam/cheetah/tree/eiger-zmq).

#### 2.3.2. Data processing using *CrystFEL*   

We used *CrystFEL* (White *et al.*, 2016 ▸) version 0.6.1 to index, integrate and merge data. Prior to data processing, PILATUS cbf files were converted to the hdf5 format. The sensor gaps were removed in this conversion. Diffraction spots were selected using the built-in zaef algorithm (Zaefferer, 2000 ▸) and indexed using *DirAx* (Duisenberg, 1992 ▸). Spot intensities were integrated using direct summation; peak intensity was estimated within a radius of two pixels, and background was estimated with radii between four and six pixels. First, we processed 200 randomly selected images and optimized their zaef parameters by maximizing the number of indexed patterns, which resulted in a threshold and minimum gradient of 50 and 20000, respectively. The zaef parameter ‘minimum SNR’ was fixed to 1. Using this result, we averaged the refined detector shifts and applied the averaged value to the sensor geometry configuration file. These parameters were then used to process all images. The intensities in the stream files were merged using the Monte Carlo method with per-frame scaling using the *process_hkl* scale.

Because all images were rotational snapshots except for the still dataset (Rs-0), Lorentz-factor correction with respect to the spindle axis was required. We applied the inverse Lorentz factor 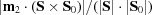, where 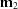 is the spindle axis (unit vector) and 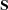 and 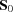 are the wavevectors of the diffracted and incident beam, respectively (Kabsch, 2010*a* ▸), to intensities and estimated errors in the stream file. Intensities were merged as described above.

#### 2.3.3. Data processing using *XDS*   

We used the custom-made script *kamo.single_images_integration* to process random snapshots using *XDS* (Kabsch, 2010*b* ▸) (version of 15 October 2015). An overview of this data processing is given in Fig. S1 of the supporting information. The images were processed individually. Indexing was performed first without using known unit-cell parameters as prior information. If indexing was not successful or not consistent with the known unit cell, then it was performed again including prior information. If the new result was not consistent with the known unit cell, the image processing was considered a failure. Next, integration by three-dimensional (3D) profile fitting was performed. All integrated intensities were written to file (MINPK=0). Because the intensities in this file were ‘full’ intensities estimated from 3D profiles, partial intensities were put aside to be stored in another file for evaluation by multiplying intensities and estimated errors by the percentage of observed reflection intensity (PEAK) as reported by *XDS*. The integrated data were subjected to a CORRECT job without any empirical correction or refinement of geometric parameters. Only non-empirical corrections including a polarization correction, and an absorption correction for air and for the Si sensor were applied in the CORRECT job. To prevent rejection of reflections where the centroids in the rotation direction were estimated to be located far away from the image number, filenames of data_10000.cbf were always used and DATA_RANGE=1 20000 was specified in CORRECT. ‘Full’ and ‘partial’ intensities were saved separately to decide later which gave a better result.

Integrated intensities (full and partial reflections separately) were merged using the custom-made script *kamo.merge_single_images_integrated*, which merged intensities and estimated the error in a Monte Carlo manner with frame scaling using the following equations as *process_hkl* in *CrystFEL* (White *et al.*, 2012 ▸),
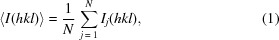


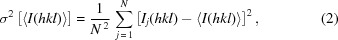
where 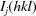 is the *j*th observation of the reflection intensity and 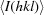 is the merged intensity obtained by Monte Carlo integration. The per-frame linear scale factor for the *i*th frame 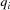 was calculated to minimize the squared residual between 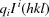 and 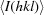, where 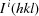 is the observed intensity for the *i*th frame. In this script, the minimum PEAK value (like MINPK= in *XDS*) can be specified. The randomly halved datasets were prepared for calculation of CC_1/2_, CC_ano_ and other parameters. The statistics were calculated using *compare_hkl* and *check_hkl* in *CrystFEL*. Note that we used *XDS* only for rotational images because *XDS* has no option to process still images (Rs-0). These custom-made scripts are available on GitHub (https://github.com/keitaroyam/yamtbx).

#### 2.3.4. Sorting of integrated results   

Individual integrated results for each image were evaluated based on 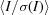 values. Note that 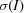 values here were derived just from counting statistics for *CrystFEL* results, whereas for *XDS* the 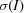 values estimated by counting statistics were modified by the default error model (*a* = 4, *b* = 10^−4^). For the stream file of *CrystFEL*, we made a script to change the chunk order based on the 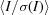 values. For *XDS* results, we sorted the list of filenames that contained the results for each image. The scripts are available on GitHub, as mentioned above.

#### 2.3.5. Anomalous difference Fourier map and SAD phasing   

Anomalous difference Fourier peak heights at mercury positions were calculated using *ANODE* (Thorn & Sheldrick, 2011 ▸). The previously solved cryogenic temperature structure of LRE (PDB code: 5GTQ) was used as the source of phases.

For SAD phasing, the *SHELX C/D/E* programs were used (versions 2013/3, 2013/2 and 2016/1, respectively) (Sheldrick, 2010 ▸). In *SHELXD*, 1000 cycles were carried out to search for the heavy atom sites, and the high-resolution cutoff was determined by *SHELXC*. In *SHELXE*, 20 cycles of density modification and polyalanine tracing were repeated 60 times. The heavy atom site located by *SHELXD* was compared with the known site in the refined structure using *phenix.emma* (Adams *et al.*, 2002 ▸) and *SHELXE* was executed only when the site was correctly located. To determine the minimum required number of images for successful Hg-SAD phasing, the above protocol was first performed for cumulative groups of 1000 images. The lowest number of unsuccessful images was then cumulatively increased by 200 images to find the required minimum.

#### 2.3.6. Structure refinement   

The previously solved LRE structure was refined against Ds-1.1^*i*th^ (*i* = 1, 2,…, 25) to evaluate the occupancies and *B*-values of the mercury atoms. Alternate conformations and waters were removed before refinement. For Ds-1.1^19th^ and later, only one mercury atom was modeled since the electron density of mercury atoms was so poor. With *phenix.refine* version 1.10.1 (Afonine *et al.*, 2012 ▸), rigid-body refinement followed by refinement of individual atomic coordinates, atomic displacement parameters (ADPs), occupancies of mercury atoms, and automatic water placement were performed. For mercury atoms, anisotropic ADPs were refined and tabulated values of 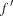 and 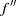 were used. The high-resolution cutoff was 1.6 Å. The 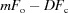 omit maps were calculated by refining the structure excluding mercury atoms.

Molecular graphics figures were prepared using *PyMOL* (The PyMOL Molecular Graphics System, Version 1.8, Schrödinger, LLC). All plots were prepared using *R* (R Development Core Team, 2008 ▸) with the *ggplot2* package (Wickham, 2009 ▸).

### 2.4. Estimation of the mosaicity of LRE microcrystals   

The mosaicity of LRE microcrystals was estimated by collecting diffraction data using the conventional rotation method. The beam size and wavelength were the same as those used during SS-ROX data collection. The photon flux was decreased to 0.5% using a 1500 µm-thick aluminium absorber. One hundred images were collected from a single-crystal using a rotation step of 0.1° and exposure time of 0.1 s. Data from ten crystals were used to calculate the average mosaicity. The data were processed using *XDS* (Kabsch, 2010*b* ▸) and the calculated average mosaicity (estimated standard deviation of the reflection range) was 0.029° (0.003° r.m.s.d.).

## 3. Results   

### 3.1. Data processing   

Datasets Rs-0.1 to Rs-2 were processed using both *CrystFEL* and *XDS*, and the results were compared to decide which program was suitable. For processing with *XDS*, we found that discarding reflections with lower partiality and using full intensities estimated by the 3D profiles resulted in higher anomalous signals (Fig. S2 of the supporting information). Generally, partiality estimation is not accurate using a single diffraction image; nonetheless, the full intensity estimation worked better than expected. The optimal partiality cutoff values depended on the rotation width; values of 30%, 50%, 70% and 80% were obtained for 0.1°, 0.25°, 0.5° and ≥1.0°, respectively. When processing rotational data using *CrystFEL*, we applied the Lorentz-factor correction with respect to the goniometer spindle axis. As evaluated using anomalous difference Fourier peak heights as a function of the number of merged images, the Lorentz-factor correction improved the data accuracy, whereas *XDS* with the optimal partiality cutoff gave a much higher anomalous signal (Fig. S3a). This could be partly because *CrystFEL* calculates predicted spot positions assuming a still snapshot with finite wavelength dispersion and beam divergence, which results in under-predicted spots and lower multiplicities for larger rotation widths than those determined by *XDS* (Fig. S3b). In contrast, for smaller rotation widths, multiplicities obtained from *CrystFEL* were higher than those obtained from *XDS*, but data quality was lower because of the inclusion of low-partiality reflections that were rejected in the processing using *XDS*. According to these results, we decided to use *XDS* to process rotational snapshots. However, Rs-0 was processed using *CrystFEL*. Datasets Da-1.2 to Da-26 and Ds-1.1^*i*th^ (*i* = 1, 2,…, 25), all of which were collected with a helical rotation step of 0.5°, were processed using *XDS* with the parameters defined for Rs-0.5.

### 3.2. Influence of helical rotation step on SS-ROX   

#### 3.2.1. Hit rate and indexing rate   

The hit rates and index rates of Rs-0 to Rs-2 are presented in Table 1 ▸. All hit rates were in the range 36% to 50%. The low hit rates of Rs-0 and Rs-0.1 were caused by the inclusion of one low-hit-rate loop on which the density of microcrystals seemed to be lower compared with that of the other loops. The hit rates calculated by excluding these data were almost the same as those of the other datasets (data not shown), which implies that the hit rate does not depend on the helical rotation step. The indexing rates of Rs-0.1 to Rs-2 ranged from 50% to 62%, and that of Rs-0 was 81%.

#### 3.2.2. Resolution limit   

**Figure 2:**
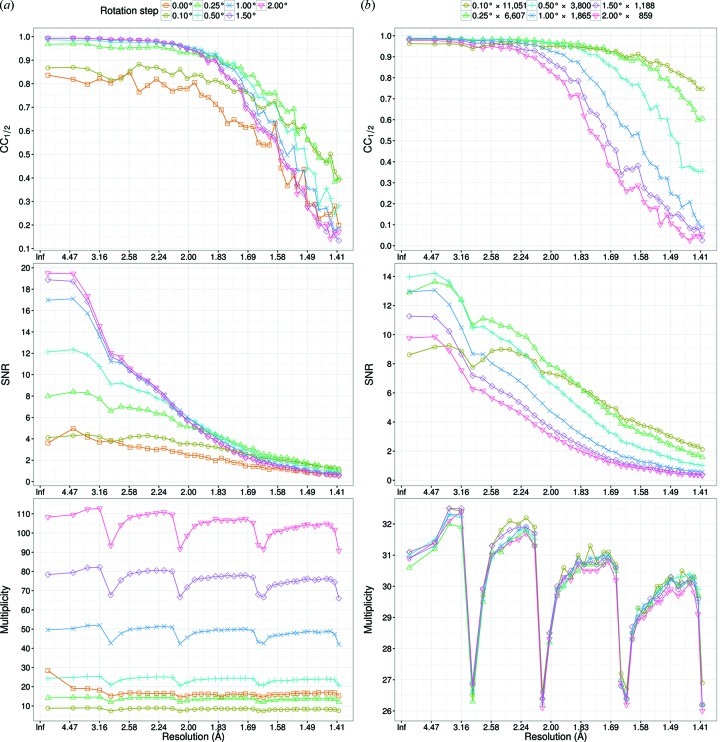
CC_1/2_, 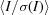 and multiplicity in each resolution shell for Rs-0 to Rs-2.0. (*a*) 3000 images were merged for each dataset. (*b*) The number of images was changed so that each dataset had almost the same multiplicity of 30. Decreases of multiplicity at resolutions of 2.9, 2.1, 1.6 and 1.4 Å were caused by dead regions of the PILATUS detector. Images were not sorted on 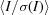 for these calculations.

The resolution was evaluated using CC_1/2_ and 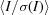 as criteria for the first 3000 images without sorting (Fig. 2*a* ▸). A plot of CC_1/2_ against resolution shows that a larger helical rotation step leads to a higher CC_1/2_ up to 2 Å. However, the trend changed beyond 2 Å; CC_1/2_ of Rs-2, Rs-1.5, Rs-1 and Rs-0.5 decreased sharply in this order. When the resolution was cut at CC_1/2_ = 0.5, the resolution limits increased in the order of Rs-2, Rs-1.5, Rs-1, Rs-0.5 and Rs-0.25. Rs-0.25 and Rs-0.1 had almost the same resolution limit, while Rs-0 had the lowest resolution of all the datasets. These results indicate that decreasing the helical rotation step is effective to improve resolution, but this effect ceases once the helical rotation step is smaller than 0.25°.

Because the exposure time per angle is larger for a smaller rotation step, higher CC_1/2_ and 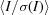 values were expected for data with a small rotation step. The inverse tendency observed in the low-resolution region might be caused by the higher multiplicity at larger rotation steps. Fig. 2(*a*) ▸ clearly shows that multiplicity increases with rotation step except for Rs-0, which was processed using *CrystFEL*. To confirm this trend, CC_1/2_ and 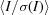 were also compared by changing the number of merged images so that all datasets had almost the same multiplicity of 30 (Fig. 2*b* ▸). The plots of CC_1/2_ and 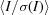 against resolution in Fig. 2(*b*) ▸ show that the difference for low-resolution shells was much smaller than that in Fig. 2(*a*) ▸, but Rs-0.1 still had the smallest CC_1/2_ value of all the datasets. This was caused by less accurate estimation of full reflection intensity by *XDS* because of the smaller partiality. The differences of CC_1/2_ and 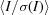 for small and large rotation data in Fig. 2(*b*) ▸ were larger than those in Fig. 2(*a*) ▸ at high resolution, which is attributed to the longer total exposure time of small rotation steps than that of large ones because of the larger number of merged images.

#### 3.2.3. Anomalous signal and Hg-SAD phasing   

**Figure 3:**
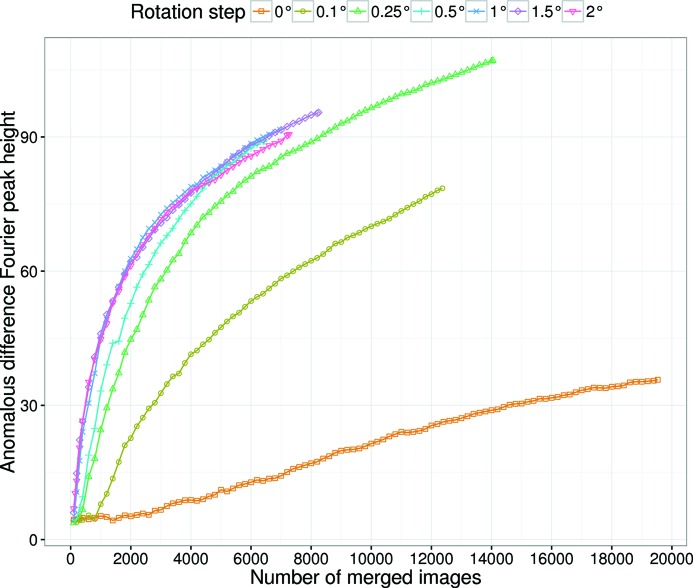
Peak heights of the anomalous difference Fourier map at the Hg site as a function of the number of merged images for Rs-0 to Rs-2. Images were not sorted on 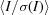 for this calculation.

The anomalous signal for Rs-0 to Rs-2 was evaluated by the peak height of the anomalous difference Fourier map as shown in Fig. 3 ▸, in which the peak height at the major site is plotted against the number of merged images. For all datasets, the anomalous peak height increased with the number of merged images, demonstrating that an increase in multiplicity improved the accuracy of the anomalous differences. The initial slopes of the curves in Fig. 3 ▸ increased with the helical rotation step up to 1°, but then remained almost constant beyond 1°. To estimate the minimum number of images required for successful Hg-SAD phasing, structure determination was attempted. The minimum number of images was 17800 for Rs-0, 3400 for Rs-0.1, 1200 for Rs-0.25, 1000 for Rs-0.5, 600 for Rs-1 and 400 for Rs-1.5 and Rs-2 (Fig. S8a). These results indicate that a helical rotation step of ≥1° is suitable for SAD phasing of LRE.

### 3.3. Influence of dose on SS-ROX   

#### 3.3.1. Hit rate and indexing rate   

The hit and indexing rates of Da-1.2 to Da-26 are presented in Table 2 ▸. The hit rates increased slightly with dose. However, because the variation was comparable with those caused by variation of sample thickness, it was difficult to confirm that the dose contributed to the increase of hit rate.

#### 3.3.2. Resolution limit   

**Figure 4:**
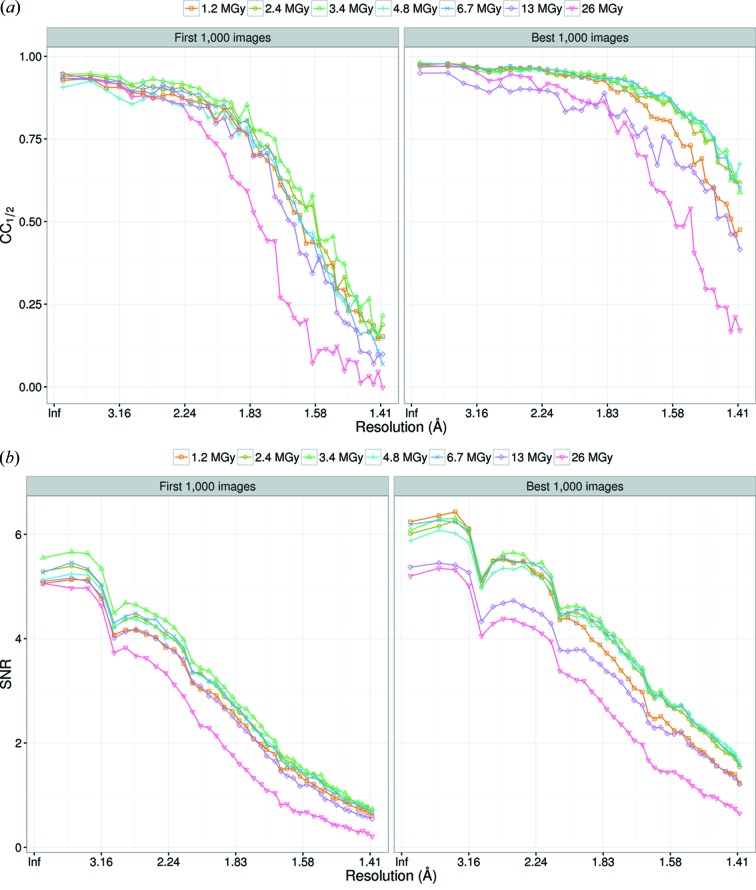
(*a*) CC_1/2_ and (*b*) 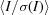 values for Da-1.2 to Da-26 as a function of resolution. The left panel of each figure was calculated by merging the first 1000 images. The right panel was prepared by merging the best 1000 images, where the best images were selected based on 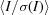 of each image.

Datasets Da-1.2 to Da-26 were collected by increasing the dose from 1.2 to 26 MGy to examine its influence on the data. Figs. 4(*a*) and 4(*b*) ▸ display CC_1/2_ and 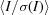, respectively, of Da-1.2 to Da-26 as a function of resolution. For both CC_1/2_ and 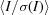, two plots are shown: one was estimated by merging the first 1000 images and the other was estimated by merging the best 1000 images based on 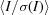 of each image. Comparison of these two plots reveals that the resolution of all datasets is improved by using the best images. Estimating the resolution limit using CC_1/2_ and 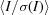 derived from the best 1000 images indicated there is a considerable increase in resolution from Da-1.2 to Da-2.4, a significant increase from Da-2.4 to Da-3.4, a marginal increase from Da-3.4 to Da-4.8, a marginal decrease from Da-4.8 to Da-6.7, and a substantial decrease from Da-6.7 to Da-26.

Datasets Ds-1.1^*i*th^ (*i* = 1, 2,…, 25) were collected by repeating the scan 25 times using the same sample with a dose of 1.1 MGy to examine the influence of sample damage on the data. Averaged integrated intensity is plotted against accumulated dose in Fig. S4. Diffraction power decreased to 70% of its initial value after accumulation of about 10 MGy, which is one-third of the Garman limit (Owen *et al.*, 2006 ▸). The plots of CC_1/2_ and 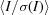 against resolution for Ds-1.1^*i*th^ (*i* = 1, 2,…, 25) using the best 1000 images in Fig. S5 show a gradual decrease of the resolution limit as the dose accumulated.

#### 3.3.3. Anomalous signal and Hg-SAD phasing   

**Figure 5:**
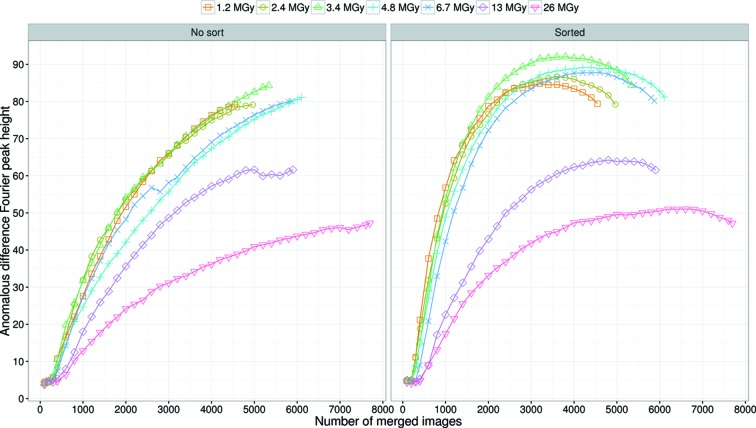
Peak heights of anomalous difference Fourier maps as a function of the number of merged images for Da-1.2 to Da-26. The left figure was prepared without 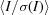 image sorting. The right figure was prepared after sorting based on 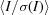 of each image.

The dose-dependent anomalous signal was evaluated by the peak height of the anomalous difference Fourier map as shown in Fig. 5 ▸, where the peak heights of Da-1.2 to Da-26 are plotted against the number of merged images with and without sorting by 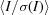. Comparison of the plots shown in Fig. 5 ▸ demonstrates that sorting made the initial slope of the plots steeper and the peak heights display a convex upward function as the number of merged images increased, indicating that inclusion of lower 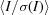 images caused the anomalous signal to deteriorate. After sorting, the initial slope of Da-1.2 was the greatest and it decreased with dose. On the other hand, the maximum peak height increased up to 3.4 MGy, and started to decrease beyond 3.4 MGy. The maximum peak heights of Da-4.8 and Da-6.7 were comparable with that of Da-3.4, whereas the peak heights of Da-13 and Da-26 were ≤75% of Da-3.4. When the images with highest 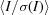 values were used, the minimum number of images required for successful Hg-SAD phasing was 600 for Da-1.2, 800 for Da-2.4, 600 for Da-3.4, 1000 for Da-4.8, 1200 for Da-6.7, 2400 for Da-13 and 3200 for Da-26 (Fig. S8b). The corresponding anomalous difference Fourier peak heights are around 50 r.m.s.d. for Da-1.2 to Da-13 and 40 r.m.s.d. for Da-26 (Fig. 5 ▸).

**Figure 6:**
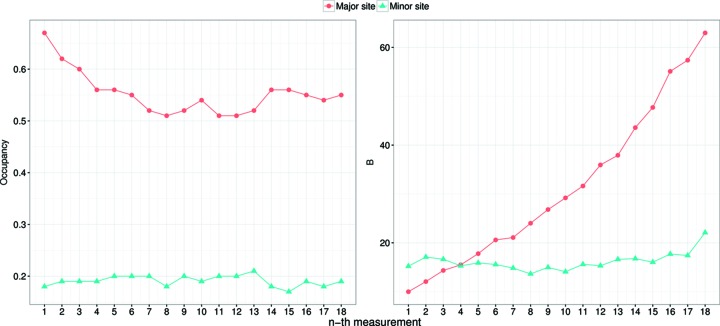
Refined occupancies and *B* values of Ds-1.1^*i*th^ (*i* = 1, 2,…, 18). All images were used for each dataset. Red circles are refined values of the major site and blue triangles are those of the minor site. The values for Ds-1.1^*i*th^ (*i* = 19, 20,…, 25) were omitted from the plot because they could not be refined properly with the same protocol that was used for the other datasets owing to the low electron density quality of mercury atoms.

**Figure 7:**
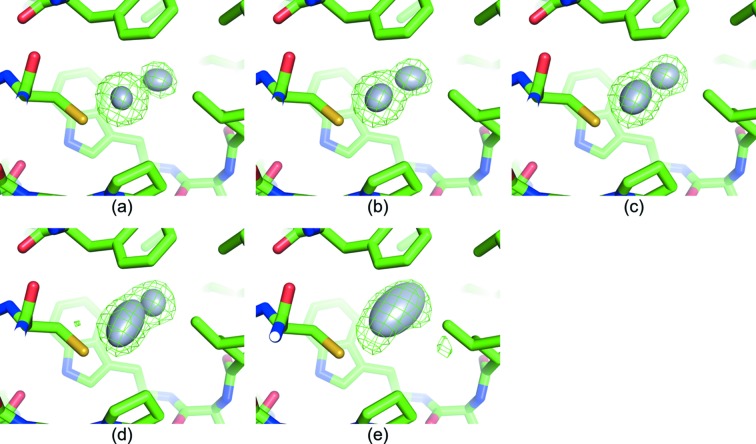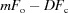 omit maps around the mercury site contoured at 5.0 r.m.s.d. (green mesh). The thermal ellipsoids of mercury atoms are drawn (probability >0.5) as grey spheroids. Yellow, red, blue and green sticks represent sulfur, oxygen, nitrogen and carbon atoms, respectively. (*a*) Ds-1.1^1st^, (*b*) Ds-1.1^3rd^, (*c*) Ds-1.1^6th^, (*d*) Ds-1.1^12th^ and (*e*) Ds-1.1^23rd^.

The peak heights of the anomalous difference Fourier maps of Ds-1.1^*i*th^ (*i* = 1, 2,…, 25) are presented in Fig. S6. Anomalous peak height gradually decreased with dose accumulation, indicating the existence of radiation damage beyond the absorption of 1.1 MGy. To examine the site-specific damage around the mercury atoms, the structural model was refined for Ds-1.1^*i*th^ (*i* = 1, 2,…, 25). The refined occupancies and atomic *B*-factor values in Fig. 6 ▸ showed a gradual decrease in occupancy and increase in *B*-factor together with increasing absorbed dose. The 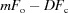 omit maps in Figs. 7 ▸ and S7 show that the electron density around the mercury atoms became more blurred with increasing accumulated dose, which reflects the decrease in occupancy and increase in *B*-factor value. These data indicate the appearance of specific damage at the mercury site with increasing dose accumulation.

## 4. Discussions   

### 4.1. Data processing in SS-ROX   

Data collected with goniometer rotation were processed using both *CrystFEL* and *XDS*. Our results revealed that *XDS* outperforms *CrystFEL* mainly because *XDS* uses a prediction model for oscillation images and discards low-partiality reflections. Because *CrystFEL* was developed to process still snapshots, it is not a suitable tool for oscillation data. In contrast, *XDS* was designed to process a series of continuous frames with increasing spindle angle rotation with a constant step, and estimated the intensity of reflections well using 3D profile fitting. We used only single frames to estimate full intensity by 3D profile fitting and merged data after applying a single-frame scale factor. Our data showed that use of single-frame 3D profile fitting gave slightly better accuracy than using observed partial intensity if the minimum partiality (PEAK) cutoff was selected properly.

A more accurate full-intensity estimation can be expected if a post-refinement program is used, *nXDS* (Kabsch, 2014 ▸) being one candidate. However, there is another difficulty in processing SS-ROX data; the diffraction image can be ‘incomplete’, meaning that the effective rotation step of a crystal can be smaller than that of the goniometer. This is because continuous translation during the helical scan can result in the crystal moving in and/or out of the incident X-ray beam. This incomplete rotation complicates post refinement, but an improvement in data accuracy can be expected if the data are treated properly. The possibility of incomplete rotation depends on the helical translation step as well as both crystal and beam size. Here a beam size comparable with the smallest dimension of the crystals was used, and all data were collected using a translation step equal to the horizontal beam size. If a smaller helical translation step is used, the possibility of incomplete rotation decreases and more accurate estimation of the diffraction intensity might be obtained.

**Figure 8:**
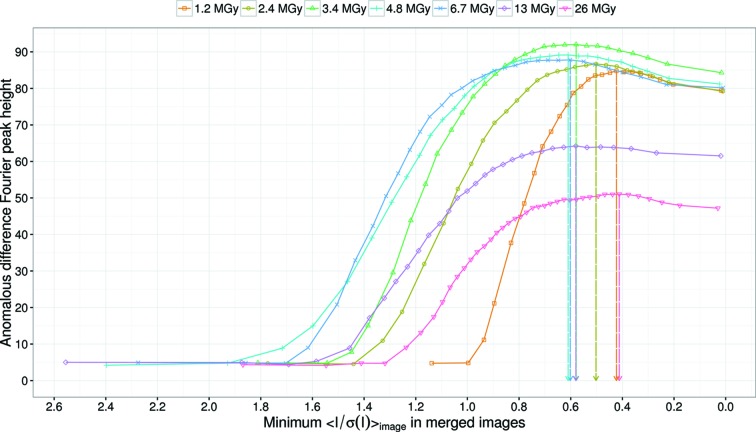
Peak heights of the anomalous difference Fourier maps of Da-1.2 to Da-26 as a function of the rejection criteria of the 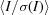 value. Vertical arrows indicate the 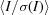 values that give the best results. Maximum peak height was observed in the 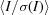 range from 0.4 to 0.6.

While processing Da-1.2 to Da-26, we noticed the importance of sorting and rejecting low-quality images prior to merging. In serial crystallography data collection, there may be variation in crystal size and quality, and there is also the possibility that only a small region of the crystal is exposed to X-ray beam. All of these factors result in variation of S/N. Inclusion of images with low S/N deteriorates the achievable resolution, as shown in Figs. 4(*a*) and 4(*b*) ▸, or results in the deterioration of the anomalous signal (Fig. 5 ▸). Therefore, rejection of lower quality data is necessary to improve the data accuracy. Here, using the 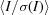 of individual images as a sorting key worked well. Our study also gave some insights into the threshold of rejection to maximize anomalous signal. Fig. 8 ▸ shows the peak height of the anomalous difference Fourier maps of Da-1.2 to Da-26 as a function of the rejection criteria of 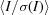. A threshold of 0.4 to 0.6 maximizes the anomalous signal.

### 4.2. Influence of the helical rotation step on SS-ROX   

Our results demonstrated that rotation was effective in SSX. In the case of LRE microcrystals, the best rotation step to achieve the highest resolution was 0.25° and a helical rotation step larger than or equal to 1° was better for Hg-SAD phasing. Both rotational steps were larger than the mosaicity of LRE microcrystals estimated by *XDS* (0.03°).

The advantages of using a fine rotation step for conventional rotation data collection methods has been examined in several studies (Pflugrath, 1999 ▸; Hülsen *et al.*, 2006 ▸; Hasegawa *et al.*, 2009 ▸; Mueller *et al.*, 2012 ▸; Casanas *et al.*, 2016 ▸). When a rotation step smaller than the reflecting range is used, one reflection is recorded over several consecutive frames, which has the advantage of decreasing background scattering. Although this method has suffered from jitter caused by inadequate shutter and goniometer control and long data collection times originating from slow detector readout, shutterless data collection using a fast-readout detector, such as a pixel array or CMOS detector, has solved these problems and a fine rotation step is now widely used. However, it has been pointed out that a smaller rotation step does not necessarily give good results. Hasegawa *et al.* (2009 ▸) showed that a rotation step that was too small was not beneficial because of the readout noise of the CMOS detector which was used. Mueller *et al.* (2012 ▸) proposed that the optimum rotation step for a PILATUS detector was half the crystal mosaicity estimated by *XDS*. Further decreasing the rotation step could reduce the accuracy because of the dead-time of the detector and/or the poor estimation of intensity from the integration software. Recently, Casanas *et al.* (2016 ▸) reported that the decrease of the rotation step down to one-tenth of mosaicity estimated by *XDS* was effective for data collection using the Eiger detector.

These differences between SS-ROX and conventional oscillation methods could be attributed to the difference of data collection and processing methods used in SS-ROX; one crystal or one region of a crystal gives only one diffraction image and the data are merged by Monte Carlo integration instead of conventional 3D profile fitting using consecutive rotational images. Therefore, in SS-ROX, the full intensity is not necessarily integrated from adjacent frames. The advantage of a larger helical rotation step in SS-ROX is wider coverage of reciprocal space, which contributes to higher multiplicity. It also contributes to recording diffraction spots as full reflections. Even when recorded as partial reflections, higher partiality is advantageous for accurate estimation of full reflection intensities by *XDS*. Conversely, a smaller rotation step is expected to have a higher S/N caused by the longer exposure time per rotation angle as well as lower background scattering. The effect of background scattering caused by sample thickness is also expected to be small when the helical rotation step is small, while large rotation increases the beam path on the sample and leads to larger background scattering. Therefore, the optimal helical rotation step in SS-ROX might be determined by a combination of these factors. Our data show that the dominant factor for determining the optimal helical rotation step differs depending on the resolution range; higher multiplicity and larger partiality were advantageous for low resolution, whereas longer exposure time per rotation angle and/or lower background scattering made important contributions at high resolution. For Hg-SAD phasing, a rotation step ≥1° was favorable in the case of LRE microcrystals. We speculate that this was because the anomalous signal was dominated by low-resolution reflections, where a larger rotation step gives higher accuracy.

As shown in Fig. S2, discarding low-partiality reflections was important. In our case, where mosaicity was 0.03°, most reflections had low partialities with 0.1° rotation, so larger rotations were beneficial to lower the fraction of low-partiality reflections. For other cases with higher mosaicity, optimal rotation could be larger and spot overlaps could be a problem. Further study using several samples is underway.

### 4.3. Influence of dose on SS-ROX   

The resolution limit of SS-ROX is assumed to be determined by the balance between the counting statistics and radiation damage. A longer exposure time decreases the counting error, but signal deterioration caused by radiation damage may occur when the exposure time is long. Even though a gradual decrease of resolution was observed beyond absorption of 1.1 MGy (Fig. S5), achievable resolution increased up to 3.4 MGy (Fig. 4 ▸) in the case of LRE microcrystals. This shows that a longer exposure time improves the S/N of weak reflections for high-resolution shells despite the existence of radiation damage.

The anomalous peak height after sorting in Fig. 5 ▸ revealed that the initial slope of the Da-1.2 plot was the steepest and gradually decreased with increasing dose. However, Da-3.4 displays the maximum peak height among the seven datasets, which was attributed to the larger S/N caused by the longer exposure time because the difference of number of merged images at the maximum peak height was small for Da-1.2, Da-2.4 and Da-3.4 (Fig. 5 ▸). Conversely, the maximum anomalous peak height began to decrease gradually beyond 3.4 MGy, implying that there was no merit in going beyond 3.4 MGy in the case of the mercury derivative of LRE. This limit will change for different heavy atom derivatives and/or proteins because the susceptibility of heavy atoms to radiation damage depends on both the type of heavy atom and its surrounding environment (Ravelli *et al.*, 2005 ▸; Ramagopal *et al.*, 2005 ▸). The deterioration of the anomalous signal at higher dose could be attributed to site-specific damage. Comparison of Ds-1.1^*i*th^ (*i* = 1, 2,…, 25) showed the propagation of site-specific damage at the mercury site (Fig. 7 ▸). Such disordering and/or partial disruption of mercury could change the relative intensity of reflections and lead to less accurate estimation of the Bijvoet differences (Owen & Sherrell, 2016 ▸).

Because the LRE microcrystals were of good quality with diffraction beyond 1.6 Å, we succeeded in SAD phasing using an exposure time of 0.02 s, which corresponds to a dose of 1.2 MGy. However, when the diffraction power was insufficient, the dose limit demonstrated here became critical; hence, data collection strategy must be considered carefully. One approach is to increase the multiplicity by using multiple samples, based on the same concept as multicrystal data collection.

## 5. Conclusion   

We have demonstrated the advantages and limitations of SS-ROX by evaluating the anomalous signal and Hg-SAD phasing of LRE. An exposure time of milliseconds to seconds at synchrotron beamlines allowed rotation during the X-ray exposure, which enabled complete data with high accuracy to be collected efficiently. Our data show that the optimum helical rotation step in SS-ROX can be determined depending on various factors such as the multiplicity and the partiality of reflections, exposure time per rotation angle and the contribution from background scattering. For the LRE microcrystals, 0.25° was the best rotation step for the achievable resolution limit, whereas a rotation larger than or equal to 1° was better for Hg-SAD phasing, where the contribution of low-resolution reflections is important. Radiation damage limited the exposure time in SS-ROX. Specific damage affecting the Hg site appeared for accumulated doses beyond 1.1 MGy, but increases in resolution and anomalous signal were observed up to 3.4 MGy because of the higher S/N of data. This limitation could be mitigated by using multiple samples. Although limited by radiation damage, we conclude that efficient data collection by SS-ROX will make it a powerful tool for protein microcrystallography.

## Supplementary Material

Supporting Figures S1 to S8. DOI: 10.1107/S1600577516016362/xh5048sup1.pdf

## Figures and Tables

**Table 1:** Hit rates and indexing rates of datasets collected with various rotation steps

Dataset	Rotation per frame ( 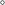 )	No. of loops	No. of collected images	No. of hit images	No. of indexed images	Hit rate (%)	Index rating (%)
Rs-0	0	5	67500	24310	19771	36.0	81.3
Rs-0.1	0.1	5	67500	24314	12366	36.0	50.9
Rs-0.25	0.25	4	54000	23745	14044	44.0	59.1
Rs-0.5	0.5	2	27000	10654	6442	39.5	60.5
Rs-1	1	2	27000	11489	6960	42.6	60.6
Rs-1.5	1.5	2	27000	13449	8248	49.8	61.3
Rs-2	2	2	27000	12456	7263	46.1	58.3

**Table 2:** Hit rates and indexing rates of datasets collected with various doses Photon flux was 2.4 × 10^12^ photons s^−1^ throughout these experiments.

Dataset	Exposure per frame (s)	Dose per frame (MGy)	No. of collected images	No. of hit images	No. of indexed images	Hit rate (%)	Indexing rate (%)	Sample thickness (mm)
Da-1.2	0.02	1.2	13500	6883	4546	51.0	33.7	0.13
Da-2.4	0.04	2.4	13500	6561	4965	48.6	36.8	0.092
Da-3.4	0.0556	3.4	13500	6846	5337	50.7	39.5	0.11
Da-4.8	0.08	4.8	13500	7575	6104	56.1	45.2	0.15
Da-6.7	0.111	6.7	13500	7371	5872	54.6	43.5	0.12
Da-13	0.222	13	13500	7240	5903	53.6	43.7	0.12
Da-26	0.435	26	13500	8509	7696	63.0	57.0	0.16

## References

[bb1] Adams, P. D., Grosse-Kunstleve, R. W., Hung, L.-W., Ioerger, T. R., McCoy, A. J., Moriarty, N. W., Read, R. J., Sacchettini, J. C., Sauter, N. K. & Terwilliger, T. C. (2002). *Acta Cryst.* D**58**, 1948–1954. 10.1107/s0907444902016657 12393927

[bb2] Afonine, P. V., Grosse-Kunstleve, R. W., Echols, N., Headd, J. J., Moriarty, N. W., Mustyakimov, M., Terwilliger, T. C., Urzhumtsev, A., Zwart, P. H. & Adams, P. D. (2012). *Acta Cryst.* D**68**, 352–367. 10.1107/S0907444912001308 PMC3322595 22505256

[bb3] Barends, T. R. M., Foucar, L., Botha, S., Doak, R. B., Shoeman, R. L., Nass, K., Koglin, J. E., Williams, G. J., Boutet, S., Messerschmidt, M. & Schlichting, I. (2014). *Nature (London)*, **505**, 244–247. 10.1038/nature12773 24270807

[bb4] Barty, A., Kirian, R. A., Maia, F. R. N. C., Hantke, M., Yoon, C. H., White, T. A. & Chapman, H. (2014). *J. Appl. Cryst.* **47**, 1118–1131. 10.1107/S1600576714007626 PMC4038800 24904246

[bb5] Botha, S., Nass, K., Barends, T. R. M., Kabsch, W., Latz, B., Dworkowski, F., Foucar, L., Panepucci, E., Wang, M., Shoeman, R. L., Schlichting, I. & Doak, R. B. (2015). *Acta Cryst.* D**71**, 387–397. 10.1107/S1399004714026327 25664750

[bb6] Boutet, S., Lomb, L., Williams, G. J., Barends, T. R. M., Aquila, A., Doak, R. B., Weierstall, U., DePonte, D. P., Steinbrener, J., Shoeman, R. L., Messerschmidt, M., Barty, A., White, T. A., Kassemeyer, S., Kirian, R. A., Seibert, M. M., Montanez, P. A., Kenney, C., Herbst, R., Hart, P., Pines, J., Haller, G., Gruner, S. M., Philipp, H. T., Tate, M. W., Hromalik, M., Koerner, L. J., van Bakel, N., Morse, J., Ghonsalves, W., Arnlund, D., Bogan, M. J., Caleman, C., Fromme, R., Hampton, C. Y., Hunter, M. S., Johansson, L., Katona, G., Kupitz, C., Liang, M., Martin, A. V., Nass, K., Redecke, L., Stellato, F., Timneanu, N., Wang, D., Zatsepin, N. A., Schafer, D., Defever, J., Neutze, R., Fromme, P., Spence, J. C. H., Chapman, H. N. & Schlichting, I. (2012). *Science*, **337**, 362–364.

[bb7] Burmeister, W. P. (2000). *Acta Cryst.* D**56**, 328–341. 10.1107/s0907444999016261 10713520

[bb8] Casanas, A., Warshamanage, R., Finke, A. D., Panepucci, E., Olieric, V., Nöll, A., Tampé, R., Brandstetter, S., Förster, A., Mueller, M., Schulze-Briese, C., Bunk, O. & Wang, M. (2016). *Acta Cryst.* D**72**, 1036–1048. 10.1107/S2059798316012304 PMC5013597 27599736

[bb9] Chapman, H. N., Fromme, P., Barty, A., White, T. A., Kirian, R. A., Aquila, A., Hunter, M. S., Schulz, J., DePonte, D. P., Weierstall, U., Doak, R. B., Maia, F. R. N. C., Martin, A. V., Schlichting, I., Lomb, L., Coppola, N., Shoeman, R. L., Epp, S. W., Hartmann, R., Rolles, D., Rudenko, A., Foucar, L., Kimmel, N., Weidenspointner, G., Holl, P., Liang, M., Barthelmess, M., Caleman, C., Boutet, S., Bogan, M. J., Krzywinski, J., Bostedt, C., Bajt, S., Gumprecht, L., Rudek, B., Erk, B., Schmidt, C., Hömke, A., Reich, C., Pietschner, D., Strüder, L., Hauser, G., Gorke, H., Ullrich, J., Herrmann, S., Schaller, G., Schopper, F., Soltau, H., Kühnel, K.-U., Messer­schmidt, M., Bozek, J. D., Hau-Riege, S. P., Frank, M., Hampton, C. Y., Sierra, R. G., Starodub, D., Williams, G. J., Hajdu, J., Timneanu, N., Seibert, M. M., Andreasson, J., Rocker, A., Jönsson, O., Svenda, M., Stern, S., Nass, K., Andritschke, R., Schröter, C.-D., Krasniqi, F., Bott, M., Schmidt, K. E., Wang, X., Grotjohann, I., Holton, J. M., Barends, T. R. M., Neutze, R., Marchesini, S., Fromme, R., Schorb, S., Rupp, D., Adolph, M., Gorkhover, T., Andersson, I., Hirsemann, H., Potdevin, G., Graafsma, H., Nilsson, B. & Spence, J. C. H. (2011). *Nature (London)*, **470**, 73–77.

[bb10] Coquelle, N., Brewster, A. S., Kapp, U., Shilova, A., Weinhausen, B., Burghammer, M. & Colletier, J.-P. (2015). *Acta Cryst.* D**71**, 1184–1196. 10.1107/S1399004715004514 PMC4427202 25945583

[bb11] Duisenberg, A. J. M. (1992). *J. Appl. Cryst.* **25**, 92–96.

[bb12] Fischetti, R. F., Xu, S., Yoder, D. W., Becker, M., Nagarajan, V., Sanishvili, R., Hilgart, M. C., Stepanov, S., Makarov, O. & Smith, J. L. (2009). *J. Synchrotron Rad.* **16**, 217–225. 10.1107/S0909049508040612 PMC2725011 19240333

[bb13] Flot, D., Mairs, T., Giraud, T., Guijarro, M., Lesourd, M., Rey, V., van Brussel, D., Morawe, C., Borel, C., Hignette, O., Chavanne, J., Nurizzo, D., McSweeney, S. & Mitchell, E. (2010). *J. Synchrotron Rad.* **17**, 107–118. 10.1107/S0909049509041168 PMC3025444 20029119

[bb14] Fukuda, Y., Tse, K. M., Nakane, T., Nakatsu, T., Suzuki, M., Sugahara, M., Inoue, S., Masuda, T., Yumoto, F., Matsugaki, N., Nango, E., Tono, K., Joti, Y., Kameshima, T., Song, C., Hatsui, T., Yabashi, M., Nureki, O., Murphy, M. E. P., Inoue, T., Iwata, S. & Mizohata, E. (2016). *Proc. Natl Acad. Sci. USA*, **113**, 2928–2933. 10.1073/pnas.1517770113 PMC4801246 26929369

[bb15] Garman, E. F. (2010). *Acta Cryst.* D**66**, 339–351. 10.1107/S0907444910008656 PMC2852297 20382986

[bb16] Gati, C., Bourenkov, G., Klinge, M., Rehders, D., Stellato, F., Oberthür, D., Yefanov, O., Sommer, B. P., Mogk, S., Duszenko, M., Betzel, C., Schneider, T. R., Chapman, H. N. & Redecke, L. (2014). *IUCrJ*, **1**, 87–94. 10.1107/S2052252513033939 PMC4062088 25075324

[bb17] González, A. (2007). *J. Synchrotron Rad.* **14**, 43–50. 10.1107/S0909049506041045 17211071

[bb18] Hasegawa, K., Hirata, K., Shimizu, T., Shimizu, N., Hikima, T., Baba, S., Kumasaka, T. & Yamamoto, M. (2009). *J. Appl. Cryst.* **42**, 1165–1175. 10.1107/S0021889809042277 PMC3246825 22477775

[bb20] Henderson, R. (1990). *Proc. R. Soc. London B*, **241**, 6–8.

[bb21] Hirata, K., Kawano, Y., Ueno, G., Hashimoto, K., Murakami, H., Hasegawa, K., Hikima, T., Kumasaka, T. & Yamamoto, M. (2013). *J. Phys. Conf. Ser.* **425**, 012002.

[bb59] Hirata, K. *et al.* (2017). In preparation.

[bb22] Holton, J. M. (2009). *J. Synchrotron Rad.* **16**, 133–142.

[bb63] Huang, C.-Y., Olieric, V., Ma, P., Howe, N., Vogeley, L., Liu, X., Warshamanage, R., Weinert, T., Panepucci, E., Kobilka, B., Diederichs, K., Wang, M. & Caffrey, M. (2016). *Acta Cryst.* D**72**, 93–112. 10.1107/S2059798315021683 PMC4756617 26894538

[bb23] Huang, C.-Y., Olieric, V., Ma, P., Panepucci, E., Diederichs, K., Wang, M. & Caffrey, M. (2015). *Acta Cryst.* D**71**, 1238–1256. 10.1107/S1399004715005210 PMC4461204 26057665

[bb24] Hülsen, G., Broennimann, C., Eikenberry, E. F. & Wagner, A. (2006). *J. Appl. Cryst.* **39**, 550–557.

[bb25] Kabsch, W. (2010*a*). *Acta Cryst.* D**66**, 133–144. 10.1107/S0907444909047374 PMC2815666 20124693

[bb26] Kabsch, W. (2010*b*). *Acta Cryst.* D**66**, 125–132. 10.1107/S0907444909047337 PMC2815665 20124692

[bb27] Kabsch, W. (2014). *Acta Cryst.* D**70**, 2204–2216. 10.1107/S1399004714013534 PMC4118830 25084339

[bb28] Kern, J., Tran, R., Alonso-Mori, R., Koroidov, S., Echols, N., Hattne, J., Ibrahim, M., Gul, S., Laksmono, H., Sierra, R. G., Gildea, R. J., Han, G., Hellmich, J., Lassalle-Kaiser, B., Chatterjee, R., Brewster, A. S., Stan, C. A., Glöckner, C., Lampe, A., DiFiore, D., Milathianaki, D., Fry, A. R., Seibert, M. M., Koglin, J. E., Gallo, E., Uhlig, J., Sokaras, D., Weng, T.-C., Zwart, P. H., Skinner, D. E., Bogan, M. J., Messerschmidt, M., Glatzel, P., Williams, G. J., Boutet, S., Adams, P. D., Zouni, A., Messinger, J., Sauter, N. K., Bergmann, U., Yano, J. & Yachandra, V. K. (2014). *Nat. Commun.* **5**, 4371.

[bb29] Kupitz, C., Basu, S., Grotjohann, I., Fromme, R., Zatsepin, N. A., Rendek, K. N., Hunter, M. S., Shoeman, R. L., White, T. A., Wang, D., James, D., Yang, J.-H., Cobb, D. E., Reeder, B., Sierra, R. G., Liu, H., Barty, A., Aquila, A. L., Deponte, D., Kirian, R. A., Bari, S., Bergkamp, J. J., Beyerlein, K. R., Bogan, M. J., Caleman, C., Chao, T.-C., Conrad, C. E., Davis, K. M., Fleckenstein, H., Galli, L., Hau-Riege, S. P., Kassemeyer, S., Laksmono, H., Liang, M., Lomb, L., Marchesini, S., Martin, A. V., Messerschmidt, M., Milathianaki, D., Nass, K., Ros, A., Roy-Chowdhury, S., Schmidt, K., Seibert, M., Steinbrener, J., Stellato, F., Yan, L., Yoon, C., Moore, T. A., Moore, A. L., Pushkar, Y., Williams, G. J., Boutet, S., Doak, R. B., Weierstall, U., Frank, M., Chapman, H. N., Spence, J. C. H. & Fromme, P. (2014). *Nature (London)*, **513**, 261–265.

[bb30] Liebschner, D., Rosenbaum, G., Dauter, M. & Dauter, Z. (2015). *Acta Cryst.* D**71**, 772–778. 10.1107/S1399004715001030 PMC4388262 25849388

[bb31] Liu, W., Wacker, D., Gati, C., Han, G. W., James, D., Wang, D., Nelson, G., Weierstall, U., Katritch, V., Barty, A., Zatsepin, N. A., Li, D., Messerschmidt, M., Boutet, S., Williams, G. J., Koglin, J. E., Seibert, M. M., Wang, C., Shah, S. T. A., Basu, S., Fromme, R., Kupitz, C., Rendek, K. N., Grotjohann, I., Fromme, P., Kirian, R. A., Beyerlein, K. R., White, T. A., Chapman, H. N., Caffrey, M., Spence, J. C. H., Stevens, R. C. & Cherezov, V. (2013). *Science*, **342**, 1521–1524. 10.1126/science.1244142 PMC3902108 24357322

[bb32] Mueller, M., Wang, M. & Schulze-Briese, C. (2012). *Acta Cryst.* D**68**, 42–56. 10.1107/S0907444911049833 PMC3245722 22194332

[bb33] Nakane, T., Song, C., Suzuki, M., Nango, E., Kobayashi, J., Masuda, T., Inoue, S., Mizohata, E., Nakatsu, T., Tanaka, T., Tanaka, R., Shimamura, T., Tono, K., Joti, Y., Kameshima, T., Hatsui, T., Yabashi, M., Nureki, O., Iwata, S. & Sugahara, M. (2015). *Acta Cryst.* D**71**, 2519–2525. 10.1107/S139900471501857X PMC4667287 26627659

[bb34] Nass, K., Meinhart, A., Barends, T. R. M., Foucar, L., Gorel, A., Aquila, A., Botha, S., Doak, R. B., Koglin, J., Liang, M., Shoeman, R. L., Williams, G., Boutet, S. & Schlichting, I. (2016). *IUCrJ*, **3**, 180–191. 10.1107/S2052252516002980 PMC4856140 27158504

[bb35] Neutze, R., Wouts, R., van der Spoel, D., Weckert, E. & Hajdu, J. (2000). *Nature (London)*, **406**, 752–757. 10.1038/35021099 10963603

[bb36] Nogly, P., James, D., Wang, D., White, T. A., Zatsepin, N., Shilova, A., Nelson, G., Liu, H., Johansson, L., Heymann, M., Jaeger, K., Metz, M., Wickstrand, C., Wu, W., Båth, P., Berntsen, P., Oberthuer, D., Panneels, V., Cherezov, V., Chapman, H., Schertler, G., Neutze, R., Spence, J., Moraes, I., Burghammer, M., Standfuss, J. & Weierstall, U. (2015). *IUCrJ*, **2**, 168–176. 10.1107/S2052252514026487 PMC4392771 25866654

[bb37] Owen, R. L., Rudiño-Piñera, E. & Garman, E. F. (2006). *Proc. Natl Acad. Sci. USA*, **103**, 4912–4917. 10.1073/pnas.0600973103 PMC1458769 16549763

[bb38] Owen, R. L. & Sherrell, D. A. (2016). *Acta Cryst.* D**72**, 388–394. 10.1107/S2059798315021555 PMC4784669 26960125

[bb39] Pflugrath, J. W. (1999). *Acta Cryst.* D**55**, 1718–1725. 10.1107/s090744499900935x 10531521

[bb40] Ramagopal, U. A., Dauter, Z., Thirumuruhan, R., Fedorov, E. & Almo, S. C. (2005). *Acta Cryst.* D**61**, 1289–1298. 10.1107/S0907444905022316 16131763

[bb41] Ravelli, R. B. & McSweeney, S. M. (2000). *Structure*, **8**, 315–328. 10.1016/s0969-2126(00)00109-x 10745008

[bb42] Ravelli, R. B. G., Nanao, M. H., Lovering, A., White, S. & McSweeney, S. (2005). *J. Synchrotron Rad.* **12**, 276–284. 10.1107/S0909049505003286 15840911

[bb43] R Development Core Team (2008). *R: A Language and Environment for Statistical Computing.* R Foundation for Statistical Computing, Vienna, Austria.

[bb44] Redecke, L., Nass, K., DePonte, D. P., White, T. A., Rehders, D., Barty, A., Stellato, F., Liang, M., Barends, T. R., Boutet, S., Williams, G. J., Messerschmidt, M., Seibert, M. M., Aquila, A., Arnlund, D., Bajt, S., Barth, T., Bogan, M. J., Caleman, C., Chao, T.-C., Doak, R. B., Fleckenstein, H., Frank, M., Fromme, R., Galli, L., Grotjohann, I., Hunter, M. S., Johansson, L. C., Kassemeyer, S., Katona, G., Kirian, R. A., Koopmann, R., Kupitz, C., Lomb, L., Martin, A. V., Mogk, S., Neutze, R., Shoeman, R. L., Steinbrener, J., Timneanu, N., Wang, D., Weierstall, U., Zatsepin, N. A., Spence, J. C., Fromme, P., Schlichting, I., Duszenko, M., Betzel, C. & Chapman, H. N. (2013). *Science*, **339**, 227–230.

[bb45] Riekel, C., Burghammer, M. & Schertler, G. (2005). *Curr. Opin. Struct. Biol.* **15**, 556–562. 10.1016/j.sbi.2005.08.013 16168633

[bb46] Schiltz, M., Dumas, P., Ennifar, E., Flensburg, C., Paciorek, W., Vonrhein, C. & Bricogne, G. (2004). *Acta Cryst.* D**60**, 1024–1031. 10.1107/S0907444904006377 15159561

[bb47] Sheldrick, G. M. (2010). *Acta Cryst.* D**66**, 479–485. 10.1107/S0907444909038360 PMC2852312 20383001

[bb48] Smith, J. L., Fischetti, R. F. & Yamamoto, M. (2012). *Curr. Opin. Struct. Biol.* **22**, 602–612. 10.1016/j.sbi.2012.09.001 PMC3478446 23021872

[bb49] Stellato, F., Oberthür, D., Liang, M., Bean, R., Gati, C., Yefanov, O., Barty, A., Burkhardt, A., Fischer, P., Galli, L., Kirian, R. A., Meyer, J., Panneerselvam, S., Yoon, C. H., Chervinskii, F., Speller, E., White, T. A., Betzel, C., Meents, A. & Chapman, H. N. (2014). *IUCrJ*, **1**, 204–212. 10.1107/S2052252514010070 PMC4107920 25075341

[bb50] Tenboer, J., Basu, S., Zatsepin, N., Pande, K., Milathianaki, D., Frank, M., Hunter, M., Boutet, S., Williams, G. J., Koglin, J. E., Oberthuer, D., Heymann, M., Kupitz, C., Conrad, C., Coe, J., Roy-Chowdhury, S., Weierstall, U., James, D., Wang, D., Grant, T., Barty, A., Yefanov, O., Scales, J., Gati, C., Seuring, C., Srajer, V., Henning, R., Schwander, P., Fromme, R., Ourmazd, A., Moffat, K., Van Thor, J. J., Spence, J. C. H., Fromme, P., Chapman, H. N. & Schmidt, M. (2014). *Science*, **346**, 1242–1246. 10.1126/science.1259357 PMC4361027 25477465

[bb51] Thorn, A. & Sheldrick, G. M. (2011). *J. Appl. Cryst.* **44**, 1285–1287. 10.1107/S0021889811041768 PMC3246834 22477786

[bb52] Weierstall, U., James, D., Wang, C., White, T. A., Wang, D., Liu, W., Spence, J. C. H., Bruce Doak, R., Nelson, G., Fromme, P., Fromme, R., Grotjohann, I., Kupitz, C., Zatsepin, N. A., Liu, H., Basu, S., Wacker, D., Won Han, G., Katritch, V., Boutet, S., Messerschmidt, M., Williams, G. J., Koglin, J. E., Marvin Seibert, M., Klinker, M., Gati, C., Shoeman, R. L., Barty, A., Chapman, H. N., Kirian, R. A., Beyerlein, K. R., Stevens, R. C., Li, D., Shah, S. T. A., Howe, N., Caffrey, M. & Cherezov, V. (2014). *Nat. Commun.* **5**, 3309. 10.1038/ncomms4309 PMC4061911 24525480

[bb53] Weik, M., Ravelli, R. B. G., Kryger, G., McSweeney, S., Raves, M. L., Harel, M., Gros, P., Silman, I., Kroon, J. & Sussman, J. L. (2000). *Proc. Natl Acad. Sci. USA*, **97**, 623–628. 10.1073/pnas.97.2.623 PMC15380 10639129

[bb54] White, T. A., Kirian, R. A., Martin, A. V., Aquila, A., Nass, K., Barty, A. & Chapman, H. N. (2012). *J. Appl. Cryst.* **45**, 335–341.

[bb55] White, T. A., Mariani, V., Brehm, W., Yefanov, O., Barty, A., Beyerlein, K. R., Chervinskii, F., Galli, L., Gati, C., Nakane, T., Tolstikova, A., Yamashita, K., Yoon, C. H., Diederichs, K. & Chapman, H. N. (2016). *J. Appl. Cryst.* **49**, 680–689. 10.1107/S1600576716004751 PMC4815879 27047311

[bb56] Wickham, H. (2009). *ggplot2: Elegant Graphics for Data Analysis.* New York: Springer.

[bb58] Yamashita, K. *et al.* (2017). Submitted.

[bb57] Yamashita, K., Pan, D., Okuda, T., Sugahara, M., Kodan, A., Yamaguchi, T., Murai, T., Gomi, K., Kajiyama, N., Mizohata, E., Suzuki, M., Nango, E., Tono, K., Joti, Y., Kameshima, T., Park, J., Song, C., Hatsui, T., Yabashi, M., Iwata, S., Kato, H., Ago, H., Yamamoto, M. & Nakatsu, T. (2015). *Sci. Rep.* **5**, 14017. 10.1038/srep14017 PMC4566134 26360462

[bb60] Zaefferer, S. (2000). *J. Appl. Cryst.* **33**, 10–25.

[bb61] Zander, U., Bourenkov, G., Popov, A. N., de Sanctis, D., Svensson, O., McCarthy, A. A., Round, E., Gordeliy, V., Mueller-Dieckmann, C. & Leonard, G. A. (2015). *Acta Cryst.* D**71**, 2328–2343. 10.1107/S1399004715017927 PMC4631482 26527148

[bb62] Zeldin, O. B., Gerstel, M. & Garman, E. F. (2013). *J. Appl. Cryst.* **46**, 1225–1230.

