# Supplementary material for: Development of a dose-limiting data collection strategy for serial synchrotron rotation crystallography
Source: J Synchrotron Radiat. 2017 Jan 1;24(Pt 1):29–41. doi: 10.1107/S1600577516016362 (PMC5182019; doi:10.1107/S1600577516016362)
Supplement: Supplementary file 1 [file s-24-00029-sup1.pdf]

JOURNAL OF SYNCHROTRON RADIATION

**Volume 24 (2017)**

**Supporting information for article:**

# Development of a dose-limiting data collection strategy for serial synchrotron rotation crystallography

**Kazuya Hasegawa, Keitaro Yamashita, Tomohiro Murai, Nipawan Nuemket, Kunio Hirata, Go Ueno, Hideo Ago, Toru Nakatsu, Takashi Kumasaka and Masaki Yamamoto**

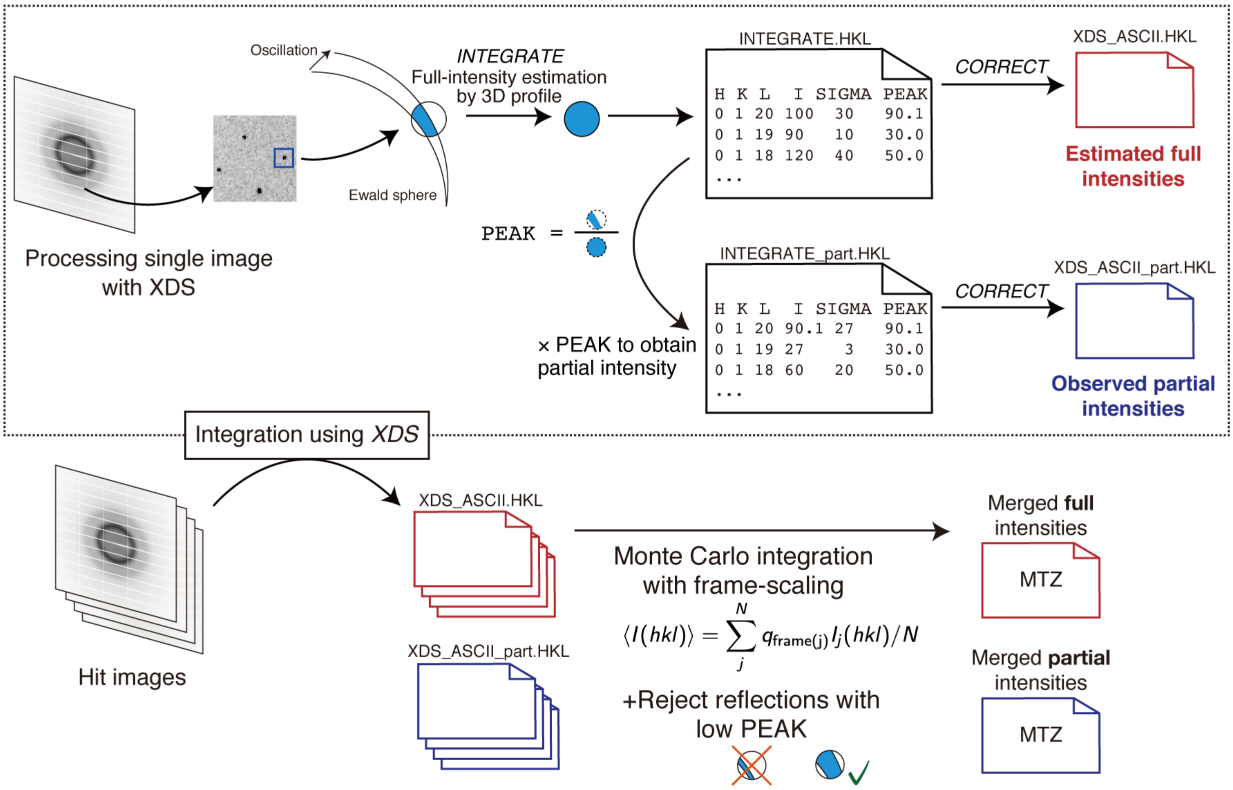


Figure S1: Data processing using *XDS*. The upper panel represents how full and partial intensities were estimated using *XDS*. The lower panel illustrates merging of integrated intensities by Monte Carlo integration.

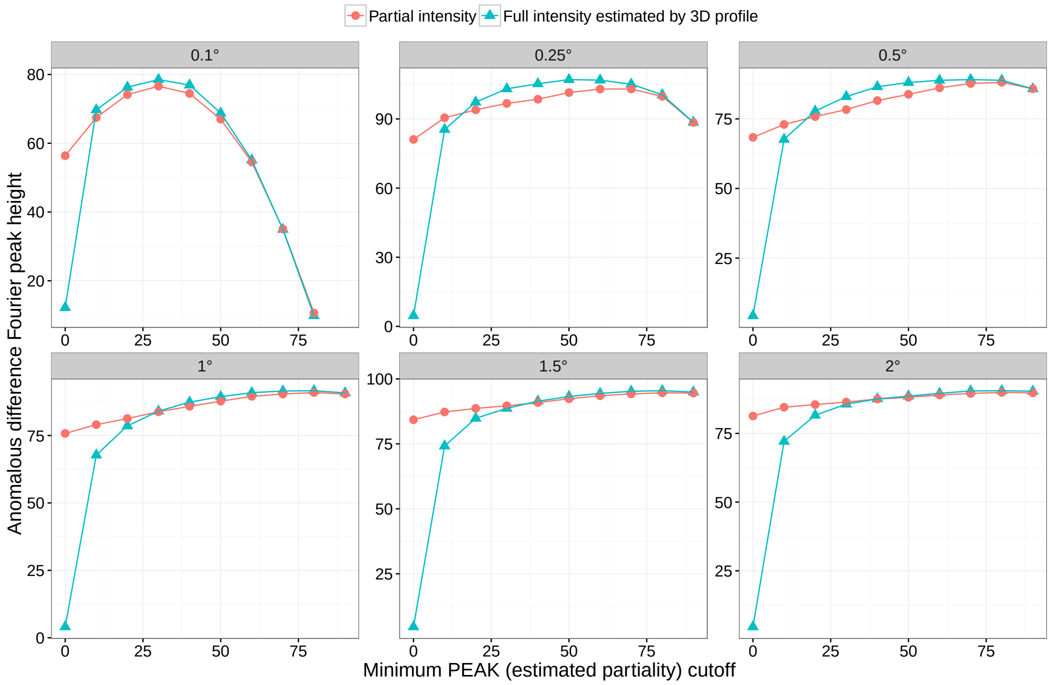


(a)

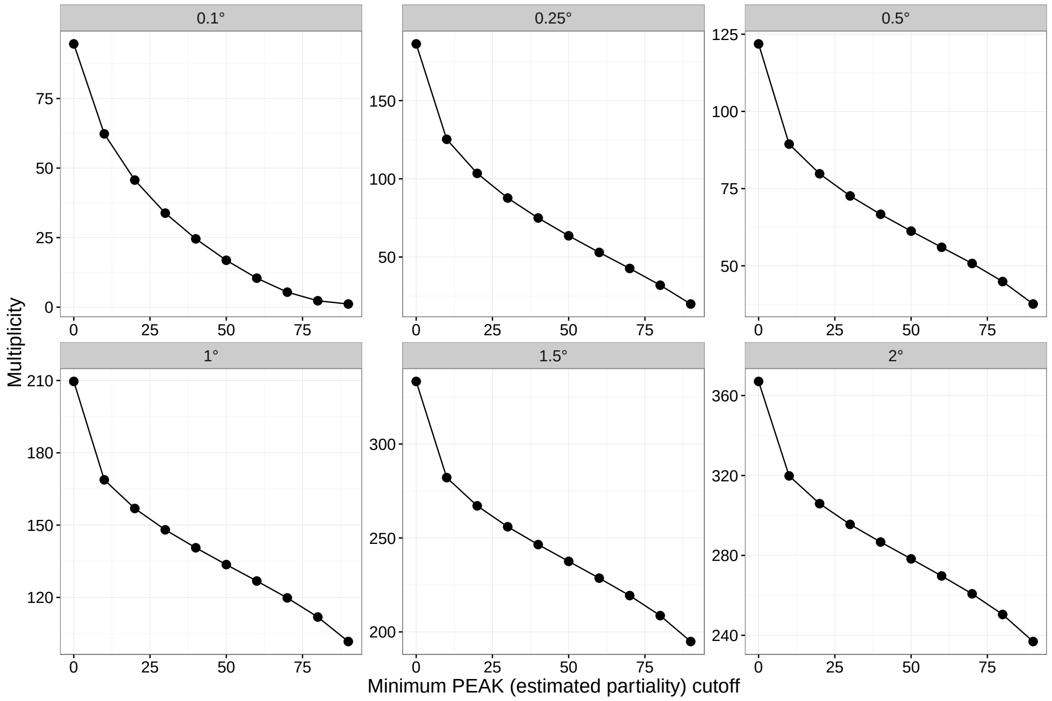


(b)

Figure S2: Decision of data processing parameters used for processing with *XDS* for Rs-0.1 to Rs-2.0. (a) Anomalous difference Fourier peak heights using all images for each rotation step as a function of minimum PEAK (estimated partiality) cutoff. Using full intensity estimation (blue triangles) resulted in a larger anomalous signal than the partial intensity calculated using the PEAK value (red circles) when the optimal minimum PEAK value was chosen. The optimal minimum PEAK value depended on the rotation step. (b) Averaged multiplicities corresponding to the datasets in (a).

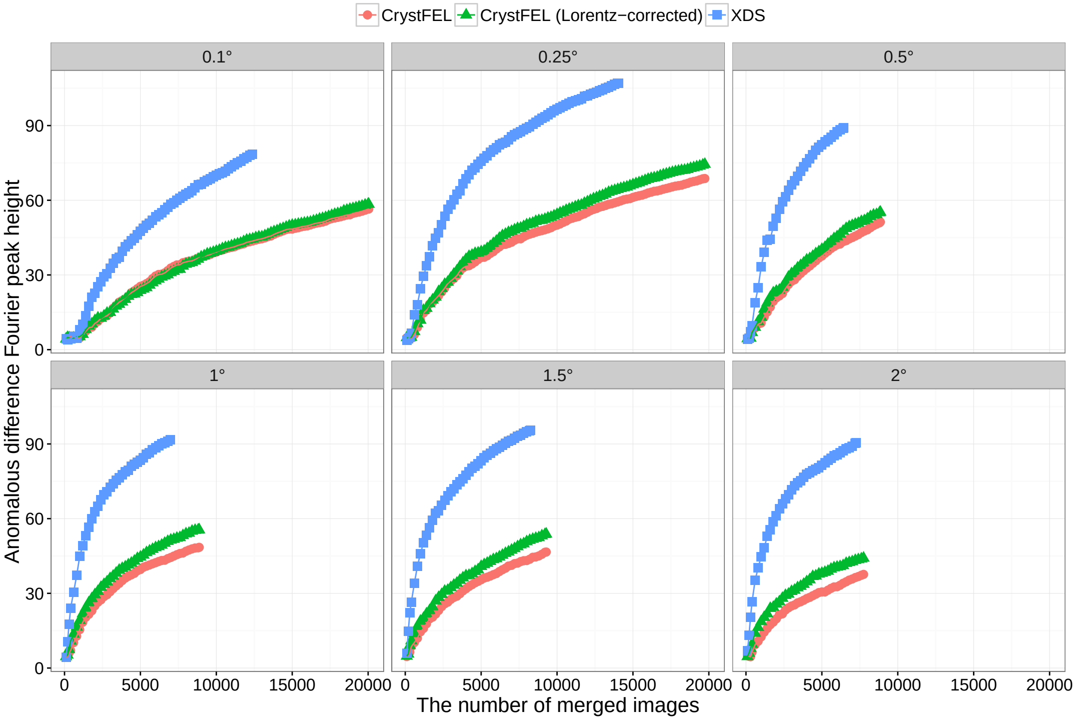


(a)

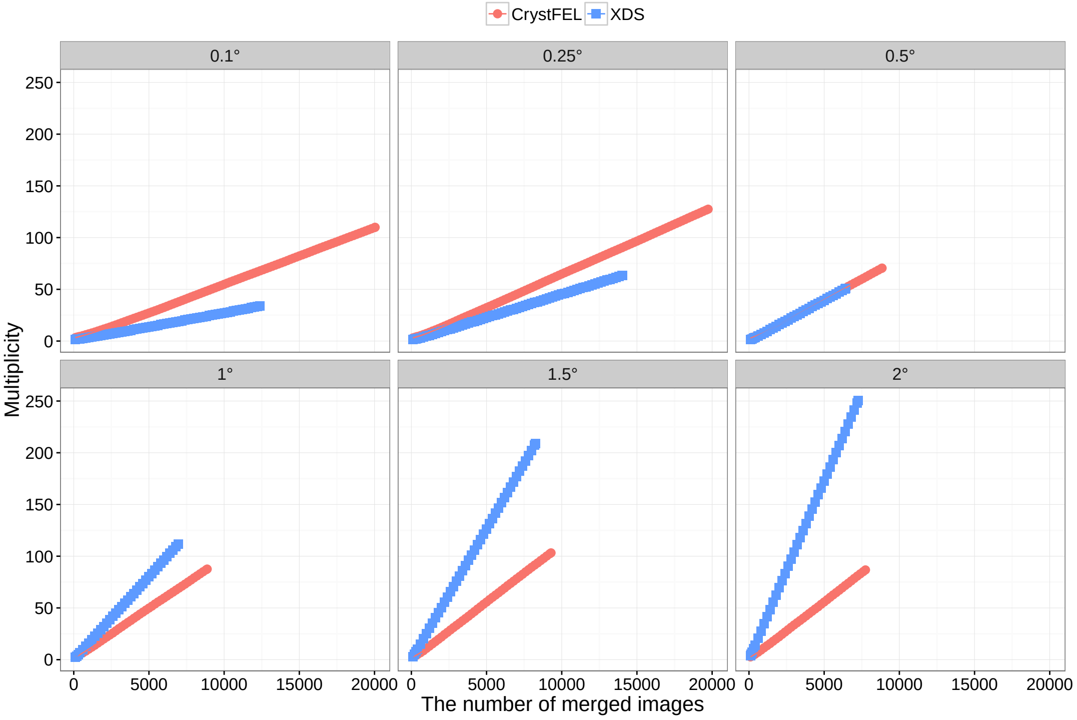


(b)

Figure S3: Comparison of *CrystFEL* and *XDS* for processing SS-ROX data (Rs-0.1 to Rs-2.0). (a) Anomalous difference Fourier peak heights and (b) averaged multiplicities as functions of the number of merged images for different data processing methods (red circles: *CrystFEL*, green triangles: *CrystFEL* with Lorentz-factor correction, blue rectangles: *XDS* with the best PEAK cutoff).

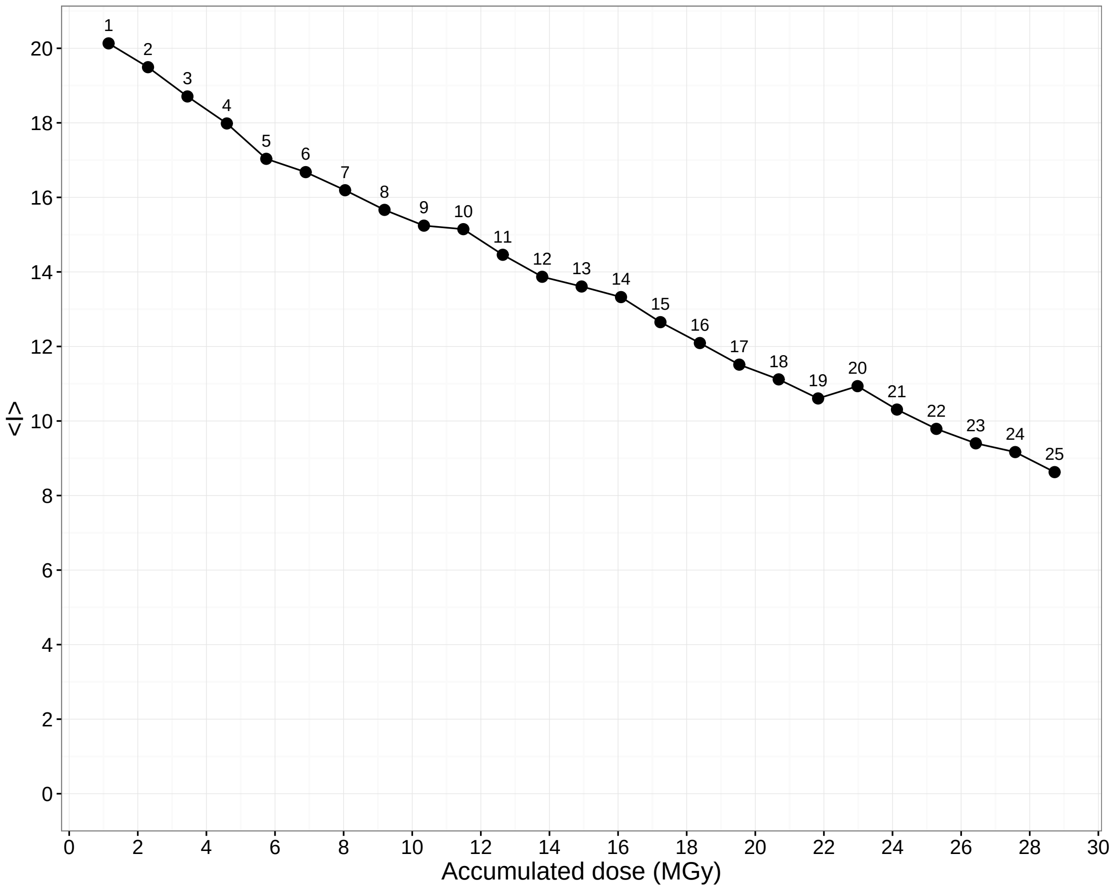


Figure S4: Diffraction power as a function of accumulation dose. Here, diffraction power was defined as the averaged integrated intensity of Ds-1.1$^{i\text{th}}$ ($i = 1, 2, ..., 25$). All reflections with a resolution of up to 1.4 Å were used for this calculation.

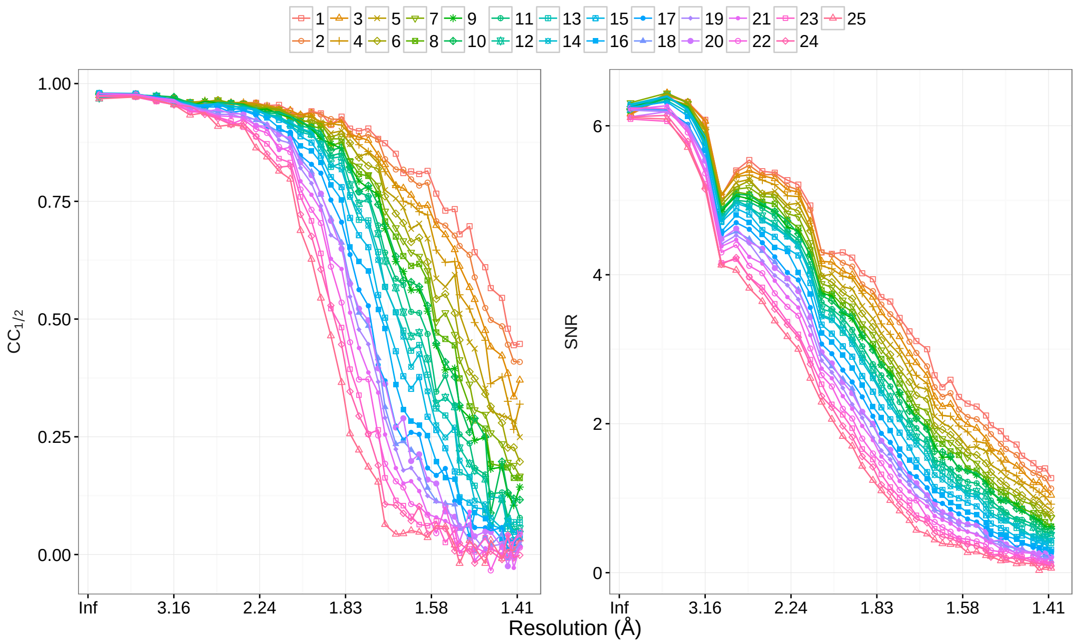


Figure S5: $CC_{1/2}$ (left) and $\langle I/\sigma(I)\rangle$ (right) of Ds-1.1$^{ith}$ ($i = 1, 2, ..., 25$) plotted as a function of resolution. The best 1,000 images selected based on $\langle I/\sigma(I)\rangle$ were used for these calculations.

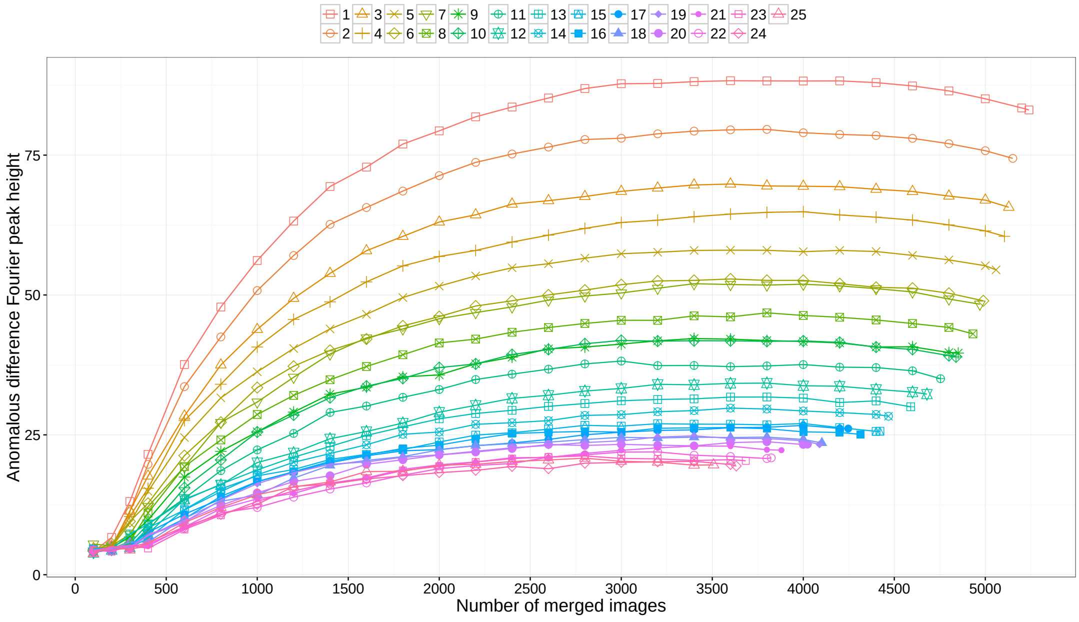


Figure S6: Peak heights of the anomalous difference Fourier maps obtained for Ds-1.1$^{ith}$ ($i = 1, 2, ..., 25$) as a function of the number of merged images. Images were sorted based on $\langle I/\sigma(I)\rangle$ before calculation.

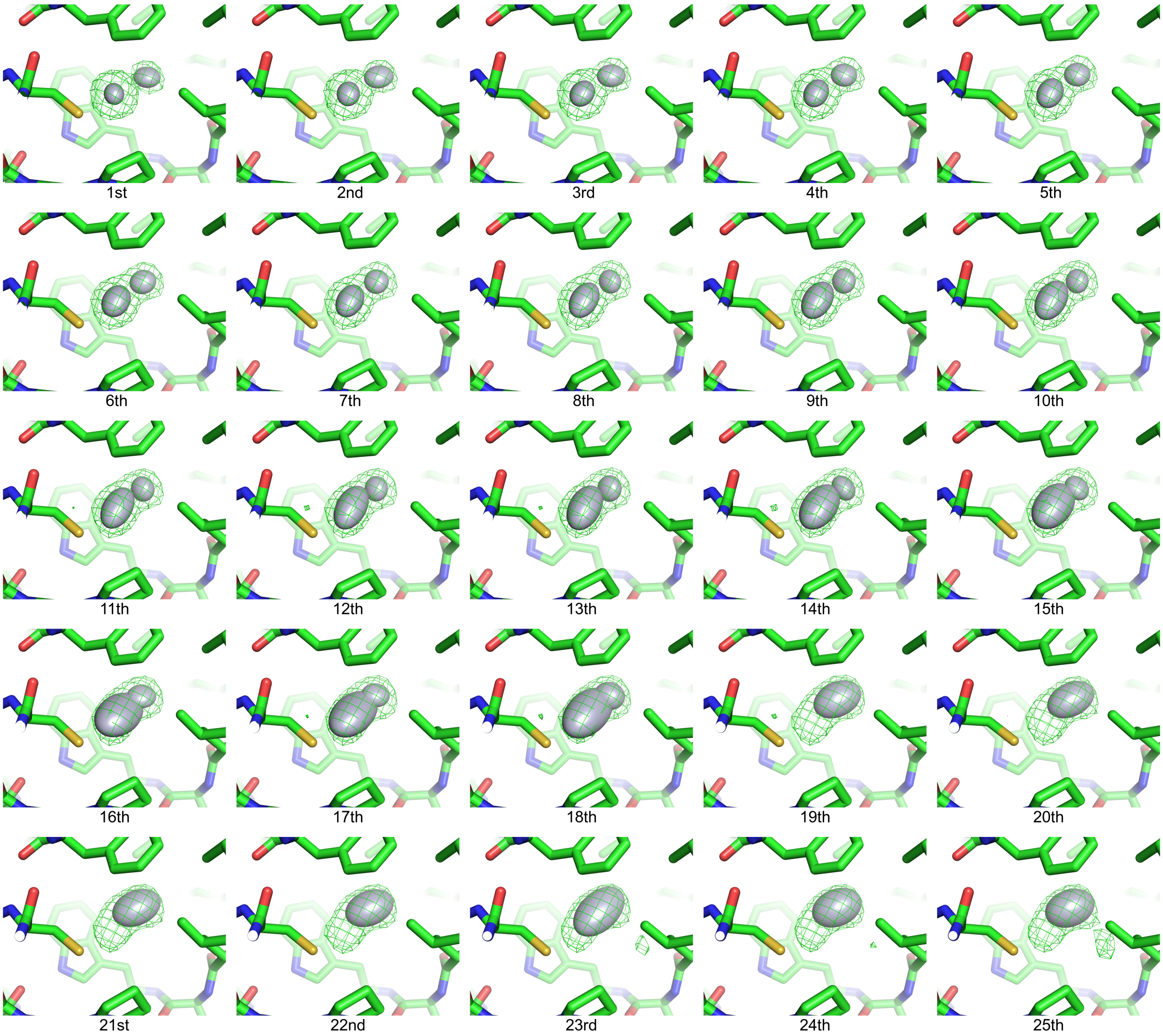


Figure S7: $mF_\mathrm{o} - DF_\mathrm{c}$ omit maps around the mercury site contoured at 5.0 rmsd for Ds-1.1$^{ith}$ ($i = 1, 2..., 25$) (green mesh). The thermal ellipsoids of mercury atoms are drawn (probability$> 0.5$). Yellow, red, blue and green sticks represent sulfur, oxygen, nitrogen and carbon atoms, respectively. For 19th and higher, the electron density of mercury atoms was so poor that only the major site was modeled.

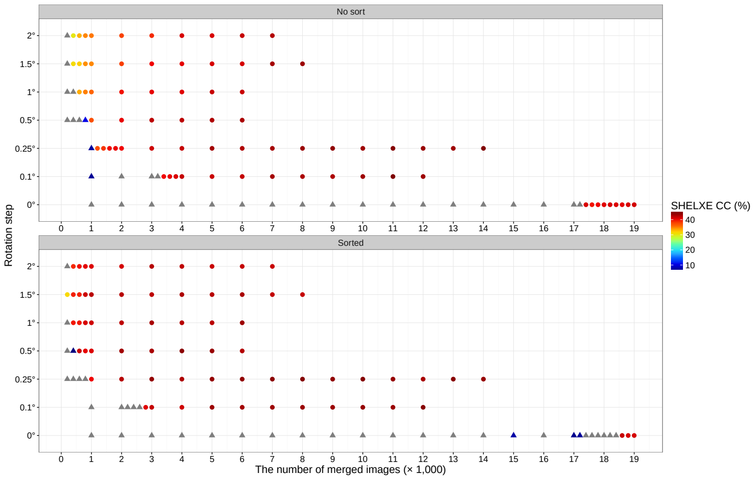


(a)

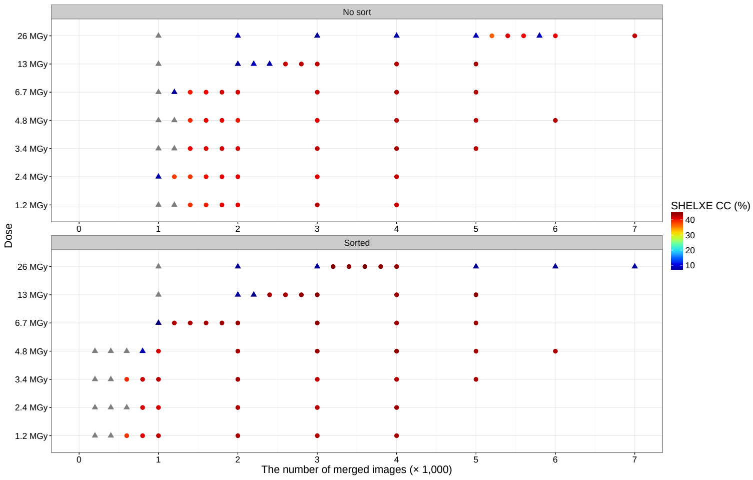


(b)

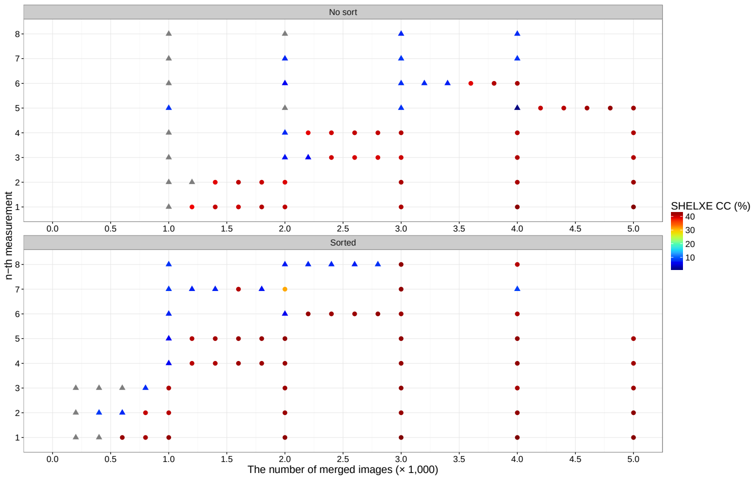


(c)

Figure S8: Hg-SAD phasing attempts using *SHELX C/D/E*. The CC values (in percentages) of traced polyalanine chains in SHELXE are shown. Results with CC values larger than 30% were judged a success (shown as circles; otherwise, triangles). Gray data points indicate failure of *SHELXE* or *SHELXD* (correct site not found). (a) Rs-0 to Rs-2.0, (b) Da-1.2 to Da-26 and (c) Ds-1.1$^{ith}$ ($i = 1$ to 8; there was no success with 9th and later. In each figure part, upper and lower panels show the results with and without sorting, respectively.
